# Management cost of acute respiratory infections in older adults in China: A systematic review and meta-analysis

**DOI:** 10.7189/jogh.14.04165

**Published:** 2024-10-11

**Authors:** Xiaoyu Xu, Tiantian Zhang, Yumeng Miao, Xiao Li, You Li

**Affiliations:** 1 Department of Epidemiology, National Vaccine Innovation Platform, School of Public Health, Nanjing Medical University, Nanjing, China; 2 Centre for Health Economics Research and Modelling Infectious Diseases (CHERMID), University of Antwerp, Belgium; 3 Centre for Global Health, Usher Institute, University of Edinburgh, Edinburgh, UK; 4 Changzhou Third People’s Hospital, Changzhou Medical Centre, Changzhou, China

## Abstract

**Background:**

Acute respiratory infection (ARI) poses a significant public health challenge worldwide, particularly among older adults. Understanding the cost of ARI management is important for optimising intervention strategy. We aimed to estimate the management cost of ARI in older adults in China.

**Methods:**

We searched three English databases (MEDLINE, Embase, and Web of Science) and four Chinese databases (Chinese National Knowledge Infrastructure, Wanfang, SinoMed, and VIP) to identify studies published between 1 January 1995 and 27 February 2023 on the cost of ARI management in older adults in China. We pooled up age group and category-specific costs across individual studies by calculating median and interquartile range (IQR). All cost results were converted and inflated to USD in 2021.

**Results:**

We included 99 studies, of which 50 were determined as high quality. In those aged >60 years, the median total cost of ARI, including direct medical, direct non-medical, and indirect cost, was USD 3263 (IQR = 2676–3786) in the inpatient setting and USD 104 (IQR = 80–129) in the outpatient setting. For both inpatient and outpatient settings, direct medical costs accounted for most of the costs (69.3% and 54.8%, respectively). There was an increasing trend over age in the median direct medical cost in the inpatient setting, ranging from USD 1517 (IQR = 1114–2017) in those aged ≥50 years to USD 3493 (IQR = 2608–4378) in those aged ≥80 years.

**Conclusions:**

Our study fills the knowledge gap on the cost of ARI and suggests that the overall cost of ARI is substantial among older adults in China. Cost data stratified by region, pathogen, and presence of comorbidities are warranted to help further identify subpopulations with higher ARI management costs.

**Registration:**

PROSPERO CRD42023485669.

Acute respiratory infection (ARI), including acute upper respiratory and lower respiratory infections, is a common respiratory infectious illness and poses a significant public health challenge worldwide. While ARI can affect individuals across all age groups, the health impact is notably more substantial among older adults. According to the results of the 2019 Global Burden of Disease study, ARI caused more than 247 million episodes of illness and more than 2.5 million deaths globally in 2019 [1], with 60% of the ARI deaths occurring in individuals aged ≥55 years. In 2019, acute lower respiratory infection was the leading cause of mortality among all communicable diseases and the fourth leading cause of mortality among all health conditions [2].

As one of the most populated countries in the world, China bears a substantial disease burden of ARI. It was estimated that about 36 million ARI episodes and 0.2 million ARI deaths occurred in China in 2019, with over half of these ARI deaths occurring in the elderly population aged ≥55 years [1]. With the ongoing ageing of the population in China [3] and no nationwide vaccination programmes for ARI among older adults, there is an expected further rise in the proportion of ARI mortality among older adults.

Over the past decade, there has been significant advancement in the development of vaccines targeting specific aetiologies of ARI suitable for older adults, notably including influenza and pneumococcal vaccines. Moreover, two respiratory syncytial virus (RSV) vaccine candidates (Arexvy and Abrysvo) met efficacy endpoints in phase three clinical trials. Recently, they received marketing approvals for administration among older adults in the USA and other high-income countries [4]. Most recently, another RSV vaccine candidate (mRNA-1345) reported favourable efficacy results in a multi-country phase two and three trial for averting RSV-associated acute lower respiratory infection among older adults [5].

Given the rapid advances in the prevention and treatment of ARI, such as new vaccines and therapeutics, it is pivotal to understand the cost of management of ARI to further support the health-economic evaluation of these new interventions. The cost of ARI management is an important driver of cost-effective analysis and has substantial impacts on the pricing strategies of products and decision-making on intervention. A recently published systematic review estimated that the global overall mean cost of ARI management in older adults was EUR 17 804 (IQR = 155–30 069) per inpatient episode, with substantial regional variations [6]. However, most of the cost data came from high-income countries, and important knowledge gaps in the cost data remain in low- and middle-income countries. To this end, we aimed to conduct a systematic review and meta-analysis on the management cost of ARI in Chinese older adults.

## METHODS

### Systematic literature search

The systematic review is registered with PROSPERO (CRD42023485669) and reported according to the Preferred Reporting Items for Systematic Reviews and Meta-Analyses (PRISMA) checklist (Table S1 in the **Online Supplementary Document**). We conducted a systematic literature search of the published literature among three English databases and four Chinese databases – MEDLINE, Embase, Web of Science, Chinese National Knowledge Infrastructure, WanFang, SinoMed, and VIP. The search terms included relevant terms on ARI, cost of management, and China. We tailored search terms to each database (Table S2 in the **Online Supplementary Document**). To ensure comprehensiveness, we used both quick search and advanced search functionalities in Chinese databases to capture all relevant records (the advanced search functionality was known to have missed some relevant records intermittently in these databases). The publication date of the literature was limited from 1 January 1995 to 27 February 2023. As this is the first systematic review of ARI management costs in older adults in China, we aimed to include as much evidence as possible while ensuring feasibility regarding the workload. Therefore, the selected publication timeframe resulted from the balance between comprehensiveness and feasibility. No language restrictions were applied to the search, although eligible reports were expected to be in English or Chinese.

### Literature screening process

We included studies that reported the real-world cost of ARI management among older adults in mainland China. As varied reporting and definitions of older adults were expected, we broadly considered any age ≥50 as older adults. We excluded non-primary studies, clinical trial studies with no standard control arms, or any other studies that did not have empirical cost data. Moreover, we excluded studies that reported only costs related to hospital-acquired ARI or focussed only on the cost of medicines, as these studies could not represent the management cost of ARI of the general population.

Based on the eligibility criteria above, two reviewers (XX and TZ) independently screened the titles and abstracts of all records retrieved from the literature search and performed full-text screening among the shortlisted records from the title and abstract screening. In addition, we manually searched the reference lists of the included studies to identify any other potentially eligible studies. Any disagreements during the screening process were discussed and resolved by consensus (where necessary, arbitrated by YL).

### Data extraction

Based on the previously published global meta-analysis of ARI management cost in older adults [6], we designed a data extraction template that consisted of two parts. In the first part, we extracted information on study characteristics, such as study period, location and region, hospital grade, study design and the perspective regarding cost. In the second part, we extracted specifically the cost-related data with one cost item per record (i.e. row), including age, gender, sample size, information on diagnosis, category of cost (direct medical costs, direct non-medical costs, indirect costs, and total costs), currency, the statistics of cost (i.e. mean, median, standard error, etc.), and the amount of cost. In China, hospitals are classified into three grades: grade one, grade two, and grade three. This is based on the size, medical technology, and breadth of hospital services, differing from the primary, secondary, and tertiary health care systems commonly referred to in Western countries. Grade one hospitals are typically smaller community or township hospitals. Grade two, larger than grade one hospitals, usually serve at the county or district level. Grade three hospitals can be large general or specialised hospitals located at the city or provincial level. We defined direct medical costs as the financial resources for health services and interventions, including expenses related to outpatient visits, hospitalisations, medications, and other costs generated during disease management. We defined direct non-medical costs as the cost of meals, transportation, accommodation, companionship, and other expenses associated with the disease. Indirect cost was defined mainly by the time and productivity lost due to disease or disease management. The data extraction process was carried out by two independent reviewers (XX and TZ) who subsequently cross-checked their findings. Any disagreements on the extracted data were discussed and resolved by consensus (where necessary, arbitrated by YL).

### Quality assessment

Two reviewers (XX and TZ) independently assessed the quality of the included studies using a modified Drummond checklist consisting of 15 questions, which primarily address methodological robustness and reporting details (Table S3 in the **Online Supplementary Document**). Any disagreements were discussed and resolved by consensus (where necessary, arbitrated by YL). Based on the assessment, we classified studies as high-quality and subsequently included them in our main analysis if they scored 10 or greater. Any studies falling below this threshold were categorised as low-quality and included only in the sensitivity analysis.

### Statistical analysis

#### Currency adjustment and conversion

For studies reporting cost data in a currency other than CNY, costs were first converted to CNY for the year of the study [7]. The costs were then adjusted to 2021 according to the annual consumer price index for health care from the National Bureau of Statistics of China [8]. Finally, all costs were converted to USD for the year 2021, based on the exchange rate (USD 1 = CNY 6.45) provided by the World Bank [9]. In cases where individual studies did not explicitly report the base year of the currency conversion, we used the median year as the base year for the adjustment.

#### Main analysis

As outlined in the quality assessment, we included high-quality studies in the main analysis. The main outcome of interest was the ARI management cost per episode by age group and cost category. This was estimated by calculating the median and interquartile range (IQR) of the reported median/mean costs (mean costs were used when available, otherwise, median costs were used) across individual studies, referred to as the median method. We also applied an alternative approach for estimating the pooled cost by calculating the sample-size weighted mean cost across the studies, referred to as the weighted mean method. The median method was chosen as the main method for its robustness against outliers, while the weighted mean method provided an alternative perspective accounting for sample size differences. We conducted Pearson correlation analysis to help compare the median and the weighted mean methods.

Moreover, when there were three or more studies with the mean and standard errors of the costs, we conducted random-effects meta-analysis to supplement the median and weighted mean methods. Although studies reporting ARI of any aetiologies were included, we excluded studies that reported the cost of management among patients with severe acute respiratory syndrome (SARS) or those with coronavirus disease 2019 (COVID-19) from the main analysis. This is because the management of SARS and COVID-19 was done according to case management regulations related to emerging infectious diseases rather than the routine route of ARI management. Such studies were included only in the subgroup analysis.

#### Subgroup analysis

As substantial heterogeneity could exist across different studies, where data allowed, we conducted subgroup analyses using the median method to understand how the cost might vary by hospital level, clinical diagnosis, region, study period, and disease severity. For the subgroup analysis on clinical diagnosis, studies that reported the cost of management among patients with SARS or COVID-19 were included.

#### Sensitivity analysis

We conducted two sensitivity analyses based on the quality assessment results. The first sensitivity analysis applied a stricter criterion, raising the cut-off for high-quality studies from 10 to 11 points. The second sensitivity analysis removed the restriction regarding the quality assessment results by including all studies regardless of quality scores.

All statistical analyses were performed using R, version 4.2.0 (R Core Team, Vienna, Austria).

#### Patient and public involvement

Patients or the public were not involved in the design, conduct, reporting or dissemination plans of this systematic review and meta-analysis.

## RESULTS

### Study characteristics

**Figure 1:**
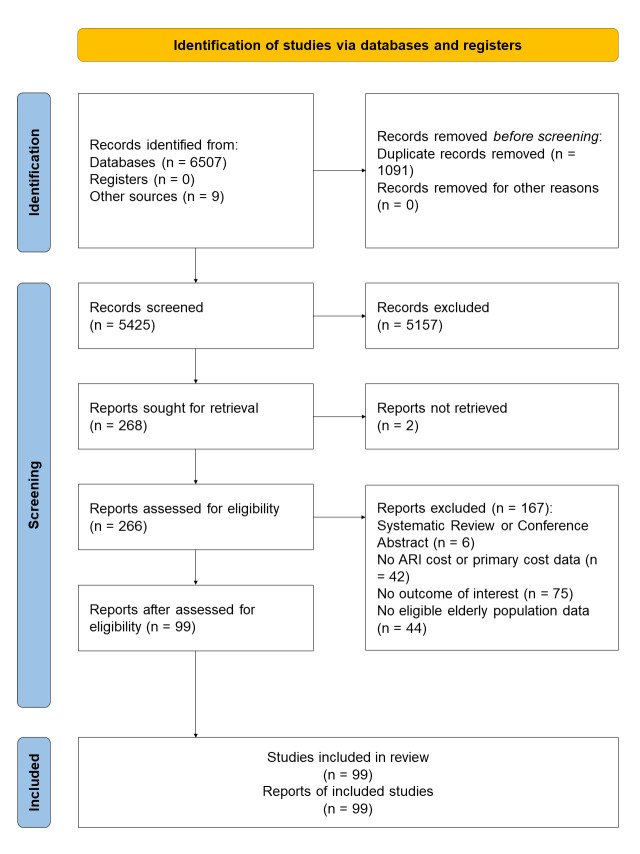
PRISMA flowchart of the study selection process.

We screened 6507 records, of which 99 studies were eligible and included in this review (**Figure 1**). The median number of ARI episodes reported per study was 73 (IQR = 40–207) (Table S4 in the **Online Supplementary Document**). These studies were from 25 out of 34 provinces (or provincial-level municipalities) in mainland China, with more than half of the studies (n = 57, 61.3%) from the eastern region of China, which is the relatively wealthier region of China (Figure S1 in the **Online Supplementary Document**). Most of the included studies were based on grade three hospitals (n = 48, 67.6%), i.e. the highest grade of hospitals in China. A high proportion of the included studies reported management costs from a hospital perspective (n = 90, 90.9%) and reported patients with community-acquired pneumonia without further restrictions to aetiologies (n = 73, 73.7%). Only a small proportion of studies (n = 11, 11.1%) reported information on the prevalence of comorbidities among ARI patients (**Table 1**).

**Table 1:** Summary of study characteristics

Study characteristics	Total reported, n (%)
China region*	
*Eastern*	57 (61.29)
*Central*	11 (11.83)
*Western*	20 (21.51)
*Northeastern*	5 (5.38)
Grade of hospital	
*Grade one*	6 (8.45)
*Grade two*	17 (23.94)
*Grade three*	48 (67.61)
Perspective	
*Societal*	9 (9.09)
*Hospital*	90 (90.91)
Study design	
*Cross-sectional*	38 (38.38)
*Retrospective cohort*	8 (8.08)
*Prospective cohort*	6 (6.06)
*Clinical trial*	33 (33.33)
*Community trial*	10 (10.10)
*Case-control*	4 (4.04)
Type of economic evaluation	
*Cost-of-illness*	54 (54.55)
*Cost-effectiveness*	45 (45.45)
Condition	
*Influenza*	13 (13.13)
*ARI*	4 (4.04)
*Community-acquired pneumonia*	73 (73.74)
*COVID-19†*	5 (5.05)
*SARS†*	4 (4.04)
Reporting of comorbidity status	
*Yes*	11 (11.11)
*No*	88 (88.89)
Quality assessment	
*High (≥10 points)*	50 (50.51)
*Low (<10 points)*	49 (49.49)
Sources of studies (databases)	
*English*	7 (7.07)
*Chinese*	93 (93.93)

ARI – acute respiratory infection, COVID-19 – coronavirus disease 2019, SARS – severe acute respiratory syndrome

*Regions were based on the economic zoning of the Chinese government.

†Included only in subgroup analysis.

### Quality assessment

According to the quality assessment using the modified Drummond checklist, approximately half of the studies (n = 50, 50.5%) [10–59] were rated as high-quality studies, with a total quality score of ≥10 points (**Table 1**). Studies receiving lower quality scores were mostly due to the lack of sensitivity analyses, inadequate cost decomposition, and incomplete information regarding cost reporting (Figure S2 and Table S5 in the **Online Supplementary Document**).

### Total management cost of ARI

Among older adults aged ≥60 years, the median total cost of ARI, including direct medical cost, direct non-medical cost, and indirect cost, was USD 3263 (IQR = 2676–3786) in the inpatient setting and USD 104 (IQR = 80–129) in the outpatient setting. For both inpatient and outpatient settings, direct medical costs accounted for most of the costs (69.3% and 54.8%, respectively), followed by indirect and direct non-medical costs. Indirect cost accounted for a larger proportion of total cost in the outpatient setting (43.0%) than in the inpatient setting (13.6%) (**Table 2**).

**Table 2:** Summary of components of cost per episode of ARI of patients aged ≥60 years in USD in 2021

	Inpatients, MD (IQR)*		Outpatients, MD (IQR)*	
**Cost**	**USD**	**%**	**USD**	**%**
Total	3263 (2676–3786)	NA	104 (80–129)	NA
Direct medical	2744 (2096–2860)	69.27 (69.18–76.68)	54 (44–63)	54.77 (51.03–58.51)
Direct non-medical	274 (232–509)	9.04 (8.72–13.16)	7 (4–9)	5.00 (3.63–6.38)
Indirect	340 (292–464)	13.64 (10.57–14.94)	46 (34–58)	43.04 (42.09–44.00)

ARI – acute respiratory infection, IQR – interquartile range, MD – median, NA – not available

*For the inpatient category three studies were included. For the outpatient category, two studies were included.

### The direct medical cost of ARI

There was an increasing trend over age in the median direct medical cost in the inpatient setting, ranging from USD 1517 (IQR = 1114–2017) in those aged ≥50 years to USD 3493 (IQR = 2608–4378) in those aged ≥80 years. Data on the direct medical cost of ARI in the outpatient setting were relatively sparse (Table 3). Sensitivity analysis that applied a higher quality threshold (i.e. ≥11 points) yielded a consistent increasing trend over age in the direct medical cost in the inpatient setting (Table S6 in the **Online Supplementary Document**). However, a similar trend was not observed in the sensitivity analysis that included all studies regardless of quality scores (Table S7 in the **Online Supplementary Document**).

**Table 3:** Summary of direct medical cost per episode of ARI in USD in 2021

Age in years	Inpatient cost in USD, MD (IQR)	Number of studies	Outpatient cost in USD, MD (IQR)	Number of studies
60–69	2434 (1633–2905)	5	72 (69–76)	2
70–79	2197 (1920–2474)	3	NA	NA
≥80	3493 (2608–4378)	2	NA	NA
≥50	1517 (1114–2017)	3	NA	NA
≥60	2077 (1158–2890)	22	54 (30–75)	4
≥65	2580 (2186–3036)	11	NA	NA
≥70	2829*	1	82 (80–85)	2

ARI – acute respiratory infection, IQR – interquartile range, MD – median, NA – not available

*We could not calculate the IQR, as we only had a single study in the analysis.

Cost estimates using the weighted mean and meta-analysis methods were generally consistent with the main method (i.e. the median method). Pearson correlation analysis showed a very high correlation between the estimates by the main method and the weighted-mean method (Pearson correlation coefficient = 0.966; *P* < 0.001) (Figure S3 in the **Online Supplementary Document**), which supported the robustness of our findings. For example, the direct medical cost of ARI for inpatients aged ≥60 years was USD 2077, USD 2128, and USD 1865 for the main, weighted-mean, and meta-analysis methods, respectively (Figures S4–15 and Table S8 in the **Online Supplementary Document**).

According to the results of the subgroup analysis, the direct medical cost for inpatients was substantially higher in those diagnosed with SARS or COVID-19 than in those diagnosed with influenza or pneumonia. Studies that included only severe ARI patients generally reported higher inpatient direct medical costs than those including both severe and non-severe patients. The inpatient direct medical cost was relatively lower in the eastern region of China than in the western and northeastern regions. No consistent trends in the inpatient direct medical cost were observed over different hospital grades or study years (**Table 4**).

**Table 4:** Summary of costs per episode of ARI of patients aged ≥60 years for subgroups in USD in 2021

Subgroup	Inpatient direct medical cost in USD, MD (IQR)	Number of studies
Hospital grade		
*Grade one*	3046 (2932–3160)	2
*Grade two*	1297 (1111–1590)	3
*Grade three*	2059 (750–2734)	11
Diagnosis		
*Influenza*	1853 (1447–2744)	5
*Pneumonia*	2059 (1060–2865)	15
*SARS*	5887 (5785–5988)	2
*COVID-19*	2532†	1
Region*		
*Eastern*	1650 (1033–2709)	14
*Western*	2306 (1775–3147)	4
*Northeastern*	2495 (2334–2656)	2
Study year		
*≤2009*	1853 (750–3036)	7
*2010–14*	2744 (1297–2938)	9
*≥2015*	2028 (1556–2459)	6
Severity of patients		
*Severe only*	2364 (2088–2824)	6
*Severe and non-severe*	1650 (987–2848)	16

COVID-19 – coronavirus disease 2019, IQR – interquartile range, MD – median, SARS – severe acute respiratory syndrome

*Regions were based on the economic zoning of the Chinese government.

†We could not calculate the IQR, as we only had a single study in the analysis.

### Duration of hospital stay

Based on 24 studies, we estimated that the median duration of hospital stays among ARI patients aged ≥60 years was 18 days (IQR = 15–19) (Table S9 in the **Online Supplementary Document**). No substantial differences were observed between different age groups.

## DISCUSSION

To the best of our knowledge, this is the first systematic review of ARI management costs among older adults in China. Our study revealed that the overall cost of ARI was substantial, with a median cost of USD 3263 (IQR = 2676–3786) per episode in the inpatient setting and USD 104 (IQR = 80–129) in the outpatient setting. Direct medical cost accounted for most of the ARI-associated cost and showed an increasing trend over age, as well as over-increased clinical severity. These findings contribute to the understanding of the economic burden of ARI in older adults in China and form an important reference for the economic evaluation of prevention and control measures against ARI.

Compared to the reported estimates of ARI management cost from a global-level meta-analysis informed mostly by data from high-income countries, the estimated cost in our study population was generally lower, particularly in the inpatient setting: USD 104 (IQR = 80–129) vs EUR 129 (IQR = 6–2494; approximately USD 153, IQR = 7–2,958) in the outpatient setting; USD 3263 (IQR = 2676–3786) vs EUR 17 804 (IQR = 155–30 069; approximately USD 21 055, IQR = 184–35 663) in the inpatient setting [6]. However, relative to the per capita disposable income of our study population, the estimated ARI management cost was substantially high. The estimated cost per inpatient episode in our study accounted for nearly 60% of the reported per capita disposable income in China in 2022 [60]. Such substantial costs could pose an extremely heavy burden to the family, especially to those without Chinese national health care insurance. Regarding the category of costs, we estimated that the average direct medical cost per ARI episode of inpatients aged ≥60 years was USD 2077 (IQR = 1158–2890), which was higher than any other cost categories. The proportion of indirect cost varied by settings. For patients aged ≥60 years, the indirect cost accounted for 13.6% of the total cost in inpatients and 43.0% in outpatients.

We observed an increasing trend over age in the direct medical cost of ARI among inpatients, consistent with studies from other countries [61,62]. However, we did not observe any substantial differences in the length of stay between different age groups, suggesting that the age-related variations in the direct medical cost were not attributable to the length of stay. We speculated that the presence of comorbidities could explain the age-related variations in the cost as the prevalence of comorbidities was expected to be higher in older patients, and management of comorbidities was associated with incremental cost [63,64]. However, no data were available in our study on the association of comorbidities with ARI management cost.

It was shown that ARI management cost was higher in less economically developed regions of China (i.e. Western and Northeastern China) – USD 2306 (IQR = 1775–3147) and USD 2495 (IQR = 2334–2656) – than the more economically developed region (i.e. Eastern China, USD 1650; IQR = 1033–2709). The variation of cost in different regions could result from different medical service costs, uses of medical services, and medical insurance. In addition, it was shown in our study that critical cases generally had a higher management cost, likely due to more frequent use of treatments such as medications, life-supporting measures such as ventilators, and longer periods of care.

We acknowledge several limitations of this study. First, for the methodological limitations, there was substantial heterogeneity among the individual studies regarding study setting, hospital grade, clinical diagnosis (e.g. ARI, pneumonia, and influenza), and threshold for admission. While we conducted subgroup analysis to understand the individual influence of these factors on the cost, we could not fully account for all of these factors simultaneously due to data scarcity. Therefore, our estimates of ARI management cost should be interpreted as a crude median cost.

Second, due to data gaps, we could not estimate the cost of ARI management according to aetiology, particularly for influenza, RSV, *Streptococcus pneumoniae*, and *Haemophilus influenzae* type B that were vaccine-preventable. Nonetheless, we did not observe substantial differences in the cost of management in our subgroup analysis by clinical diagnosis. This suggests that economic evaluation, such as cost-effectiveness analysis, might consider using the cost of ARI management as an alternative to the pathogen-specific cost data.

Third, we did not identify any studies that reported the additional cost after discharge or outpatient consultation. As a result, the reported cost of this analysis was underestimated, particularly for hospitalised patients. As hospitals in China need to retain a high bed turnover rate, patients who were discharged might not recover fully and would still need treatment. More studies that conduct follow-ups on ARI patients are warranted to fully understand the economic and health impact of ARI for the entire course of the disease. No studies reported the cost in the emergency department setting. Moreover, we did not identify any studies that reported cost from a patient perspective, which is important for assessing health care accessibility and equity, nor did we have data on the reimbursement rate for the direct medical cost or the health care coverage. The out-of-pocket cost might also be an important driver for an individual’s willingness to receive vaccination.

## CONCLUSIONS

In summary, our study provides an overview of the cost of ARI management among older adults in China. The overall estimate is well supported by the 50 high-quality studies included in this systematic review. Our findings provide important evidence for the planning of immunisation programs and health insurance policies to optimise resource allocation and health care strategies for ARIs among older adults in China. While some of the knowledge gaps are addressed by this study, the substantial remaining data gaps are worth highlighting, including the lack of cost data by region, pathogen, and comorbidities and the absence of cost data for the entire disease course in the emergency department setting, and from a patient perspective. Addressing these data gaps would further support health economic evaluation analysis to quantify the net benefits of interventions against ARI in older adults.

## Additional material


Online Supplementary Document
