# Supplementary material for: Management cost of acute respiratory infections in older adults in China: A systematic review and meta-analysis
Source: J Glob Health. 2024 Oct 11;14:04165. doi: 10.7189/jogh.14.04165 (PMC11466502; doi:10.7189/jogh.14.04165)
Supplement: Online Supplementary Document [file jogh-14-04165-s001.pdf]

Supplemental Material

Title: Management cost of acute respiratory infections in older adults in China: a systematic review and meta-analysis

Xiaoyu Xu, Tiantian Zhang, Yumeng Miao, Xiao Li, You Li

Table of Contents

Table S1. Preferred Reporting Items for Systematic Reviews and Meta-Analyses (PRISMA) checklist 3
Table S2. Search strategy 7
Table S3. Quality Assessment Criteria 10
Table S4. Summary of basic characteristics of the studies included in the review 11
Table S5. Quality and Potential for Bias 19
Table S6. Sensitivity analysis 1 23
Table S7. Sensitivity analysis 2 24
Table S8. Summary of weighted mean direct medical cost per episode of ARI in USD of 2021 25
Table S9. Summary of Summary of median (IQR) Length of Stay (LoS) per episode of ARI 26
Figure S1. Geographical distribution of included studies by province 27
Figure S2. Histogram of the distribution of original study quality scores 28
Figure S3. Forest plot of direct medical cost per ARI episode of inpatients aged 60 or more 29
Figure S4. Forest plot of direct medical cost per ARI episode of inpatients aged 65 or more 30
Figure S5. Forest plot of direct medical cost per ARI episode of inpatients of hospital grade II 31
Figure S6. Forest plot of direct medical cost per ARI episode of inpatients of hospital grade III 32
Figure S7. Forest plot of direct medical cost per ARI episode of inpatients of pneumonia 33
Figure S8. Forest plot of direct medical cost per ARI episode of inpatients of eastern China 34
Figure S9. Forest plot of direct medical cost per ARI episode of inpatients of western China 35
Figure S10. Forest plot of direct medical cost per ARI episode of inpatients of study year before 2009 36
Figure S11. Forest plot of direct medical cost per ARI episode of inpatients of study year 2010-2014 37
Figure S12. Forest plot of direct medical cost per ARI episode of inpatients of study year after 2015 38

Figure S13. Forest plot of direct medical cost per ARI episode of inpatients of critical patients only 39
Figure S14. Forest plot of direct medical cost per ARI episode of inpatients of critical and noncritical patients 40
Figure S15. Correlation of results from median and weighted mean 41
Supplementary Material References 42

Table S1. Preferred Reporting Items for Systematic Reviews and Meta-Analyses (PRISMA) checklist

Section and Topic	Item #	Checklist item	Location where item is reported
**TITLE**			
Title	1	Identify the report as a systematic review.	Title Page
**ABSTRACT**			
Abstract	2	See the PRISMA 2020 for Abstracts checklist.	P2-3
**INTRODUCTION**			
Rationale	3	Describe the rationale for the review in the context of existing knowledge.	P4-5
Objectives	4	Provide an explicit statement of the objective(s) or question(s) the review addresses.	P5
**METHODS**			
Eligibility criteria	5	Specify the inclusion and exclusion criteria for the review and how studies were grouped for the syntheses.	P6
Information sources	6	Specify all databases, registers, websites, organisations, reference lists and other sources searched or consulted to identify studies. Specify the date when each source was last searched or consulted.	P6
Search strategy	7	Present the full search strategies for all databases, registers and websites, including any filters and limits used.	P6
Selection process	8	Specify the methods used to decide whether a study met the inclusion criteria of the review, including how many reviewers screened each record and each report retrieved, whether they worked independently, and if applicable, details of automation tools used in the process.	P6-7
Data collection process	9	Specify the methods used to collect data from reports, including how many reviewers collected data from each report, whether they worked independently, any processes for obtaining or confirming data from study investigators, and if applicable, details of automation tools used in the process.	P7-8
Data items	10a	List and define all outcomes for which data were sought. Specify whether all results that were compatible with each outcome domain in each study were sought (e.g. for all measures, time points, analyses), and if not, the methods used to decide which results to collect.	P8

Section and Topic	Item #	Checklist item	Location where item is reported
	10b	List and define all other variables for which data were sought (e.g. participant and intervention characteristics, funding sources). Describe any assumptions made about any missing or unclear information.	P8
Study risk of bias assessment	11	Specify the methods used to assess risk of bias in the included studies, including details of the tool(s) used, how many reviewers assessed each study and whether they worked independently, and if applicable, details of automation tools used in the process.	P8-9
Effect measures	12	Specify for each outcome the effect measure(s) (e.g. risk ratio, mean difference) used in the synthesis or presentation of results.	
Synthesis methods	13a	Describe the processes used to decide which studies were eligible for each synthesis (e.g. tabulating the study intervention characteristics and comparing against the planned groups for each synthesis (item #5)).	P9-10
	13b	Describe any methods required to prepare the data for presentation or synthesis, such as handling of missing summary statistics, or data conversions.	P9-10
	13c	Describe any methods used to tabulate or visually display results of individual studies and syntheses.	
	13d	Describe any methods used to synthesize results and provide a rationale for the choice(s). If meta-analysis was performed, describe the model(s), method(s) to identify the presence and extent of statistical heterogeneity, and software package(s) used.	P9-10
	13e	Describe any methods used to explore possible causes of heterogeneity among study results (e.g. subgroup analysis, meta-regression).	P10-11
	13f	Describe any sensitivity analyses conducted to assess robustness of the synthesized results.	P10-11
Reporting bias assessment	14	Describe any methods used to assess risk of bias due to missing results in a synthesis (arising from reporting biases).	
Certainty assessment	15	Describe any methods used to assess certainty (or confidence) in the body of evidence for an outcome.	
**RESULTS**			

Section and Topic	Item #	Checklist item	Location where item is reported
Study selection	16a	Describe the results of the search and selection process, from the number of records identified in the search to the number of studies included in the review, ideally using a flow diagram.	P12
	16b	Cite studies that might appear to meet the inclusion criteria, but which were excluded, and explain why they were excluded.	
Study characteristics	17	Cite each included study and present its characteristics.	Table S4
Risk of bias in studies	18	Present assessments of risk of bias for each included study.	Table S5
Results of individual studies	19	For all outcomes, present, for each study: (a) summary statistics for each group (where appropriate) and (b) an effect estimate and its precision (e.g. confidence/credible interval), ideally using structured tables or plots.	
Results of syntheses	20a	For each synthesis, briefly summarise the characteristics and risk of bias among contributing studies.	
	20b	Present results of all statistical syntheses conducted. If meta-analysis was done, present for each the summary estimate and its precision (e.g. confidence/credible interval) and measures of statistical heterogeneity. If comparing groups, describe the direction of the effect.	P13-14
	20c	Present results of all investigations of possible causes of heterogeneity among study results.	P14
	20d	Present results of all sensitivity analyses conducted to assess the robustness of the synthesized results.	P14
Reporting biases	21	Present assessments of risk of bias due to missing results (arising from reporting biases) for each synthesis assessed.	
Certainty of evidence	22	Present assessments of certainty (or confidence) in the body of evidence for each outcome assessed.	
**DISCUSSION**			
Discussion	23a	Provide a general interpretation of the results in the context of other evidence.	P14-15

Section and Topic	Item #	Checklist item	Location where item is reported
	23b	Discuss any limitations of the evidence included in the review.	P16-17
	23c	Discuss any limitations of the review processes used.	P16-17
	23d	Discuss implications of the results for practice, policy, and future research.	P17-18
**OTHER INFORMATION**			
Registration and protocol	24a	Provide registration information for the review, including register name and registration number, or state that the review was not registered.	P6
	24b	Indicate where the review protocol can be accessed, or state that a protocol was not prepared.	P6
	24c	Describe and explain any amendments to information provided at registration or in the protocol.	
Support	25	Describe sources of financial or non-financial support for the review, and the role of the funders or sponsors in the review.	P19
Competing interests	26	Declare any competing interests of review authors.	P19
Availability of data, code and other materials	27	Report which of the following are publicly available and where they can be found: template data collection forms; data extracted from included studies; data used for all analyses; analytic code; any other materials used in the review.	P19

Table S2. Search strategy

Table S2-a Medline (via the Ovid interface)

#	Search Term (s)
1	exp Respiratory Tract Infections/ or respiratory tract*.mp. or exp Respiratory Tract Diseases/ or respiratory disease*.mp.
2	exp Costs and Cost Analysis/ or exp Cost of Illness/ or exp Cost-Benefit Analysis/ or cost*.mp. or econ*.mp.
3	exp Fees and Charges/ or exp Financing, Government/ or exp Financing, Personal/ or exp Health Expenditures/ or charge*.mp. or financing*.mp. or expenditure*.mp. or burden*.mp.
4	exp China/ or China.mp. or Chinese.mp.
5	2 or 3
6	1 and 4 and 5
7	limit 6 to (humans and yr="1995 - 2022" and ("middle aged (45 plus years)" or "all aged (65 and over)" or "aged (80 and over)"))

Table S2-b Embase (via the Ovid interface)

#	Search Term (s)
1	exp Respiratory Tract Infections/ or respiratory tract*.mp. or exp Respiratory Tract Diseases/ or respiratory disease*.mp.
2	exp "Costs and Cost Analysis"/ or exp "Cost of Illness"/ or exp Cost-Benefit Analysis/ or cost*.mp. or econ*.mp.
3	exp "Fees and Charges"/ or exp "Financing, Government"/ or exp "Financing, Personal"/ or exp "Health Expenditures"/ or charge*.mp. or financing*.mp. or expenditure*.mp. or burden*.mp.
4	exp China/ or China.mp. or Chinese.mp.
6	2 or 3
7	1 and 4 and 5
8	limit 6 to (human and yr="1995 - 2022" and (adult <18 to 64 years> or aged <65+ years>))

Table S2-c Web of Science

#	Search Term (s)
1	TS=(acute respiratory)
2	TS=(cost*) OR TS=(econ*)
3	TS=(charge*) OR TS=(financing*) OR TS=(expenditure*) OR TS=(burden*)
4	TS=(China) OR TS=(Chinese)
5	TS=(elder) OR TS=(elderly) OR TS=(older adults)
6	2 or 3
7	1 and 4 and 5 and 6
8	limit 7 to (yr="1995-01-01 – 2022-12-31")

Table S 2-d CNKI

#	Search Term (s)
1	(主题:急性呼吸道感染(精确))OR(篇关摘:急性呼吸道感染(模糊))OR(主题:呼吸道感染(精确))OR(篇关摘:呼吸道感染(模糊))OR(主题:呼吸道疾病(精确))OR(篇关摘:呼吸道疾病(模糊))OR(主题:肺炎(精确))OR(篇关摘:肺炎(模糊))OR(主题:细支气管炎(精确))OR(篇关摘:细支气管炎(模糊))
2	(主题:负担(精确))OR(篇关摘:负担(模糊))OR(主题:经济负担(精确))OR(篇关摘:经济负担(模糊))OR(主题:卫生经济(精确))OR(篇关摘:卫生经济(模糊))OR(主题:花费(精确))OR(篇关摘：花费(模糊))OR(主题:医疗费用(精确))OR(篇关摘:医疗费用(模糊))
3	(主题:老年人(精确))OR(篇关摘:老年人(模糊))OR(主题:老年患者(精确))OR(篇关摘:老年患者(模糊))
4	1 AND 2 AND 3
5	Limit 4 to (1995/01/01-2022/12/31)

Table S2-e SinoMed

#	Search Term (s)
1	"急性呼吸道感染"[全部字段:智能] OR "呼吸道感染"[全部字段:智能] OR "呼吸道疾病"[常用字段:智能] OR "肺炎"[常用字段:智能] OR "细支气管炎"[常用字段:智能]
2	"负担"[全部字段:智能] OR "经济负担"[全部字段:智能] OR "卫生经济"[全部字段:智能] OR "花费"[全部字段:智能] OR "医疗费用"[全部字段:智能]
3	"老年人"[全部字段:智能] OR "老年患者"[全部字段:智能]
4	1 AND 2 AND 3
5	Limit 4 to (1995-2022)

Table S2-f WANFANG DATA

#	Search Term (s)
1	全部:(急性呼吸道感染) or 全部:(呼吸道感染) or 全部:(呼吸道疾病) or 全部:(肺炎) or 全部:(细支气管炎)
2	全部:(负担) or 全部:(经济负担) or 全部:(卫生经济) or 全部:(花费) or 全部:(医疗费用)
3	全部:(老年人) or 全部:(老年患者)
4	1 AND 2 AND 3
5	Limit 4 to (1995-2022)

Table S2-g VIP

#	Search Term (s)
1	任意字段=呼吸道感染 or 呼吸道疾病 or 肺炎 or 细支气管炎
2	任意字段=负担 or 卫生经济 or 花费 or 医疗费用
3	任意字段=老年
4	1 AND 2 AND 3

5	Limit 4 to (1995-2022)

Table S3. Quality Assessment Criteria

NO.	Criteria	Yes	No	NA
1	The question and perspective are clearly stated			
2	It is a cost-study			
3	Quantity of resources used and unit costs are reported separately			
4	Data sources are clearly reported			
5	Currency and price (including price year) are documented			
6	Time horizon of costs is stated			
7	Consideration of discounting is documented and justified			
8	Details of statistical tests and result errors are clearly described			
9	Sensitivity analysis is carried out (and adjustments stated)			
10	A comparison is made between two alternatives			
11	A standard definition or diagnosis of ARI is used			
12	The answer to the study question is clearly stated (and valid)			
13	Conclusions are drawn and relevant limitations are raised			
14	Whether costs are sufficiently factored			
15	Whether the method taken is well explained			

One point for Yes, zero point for No or NA, out of a total of fifteen points. Studies achieved ten points or higher were classified as high quality, otherwise low quality, in main analysis.
ARI: acute respiratory infection; NA: not applicable.

Table S4. Summary of basic characteristics of the studies included in the review.

Author Year	Region	Grade of Hospital	Perspective	Study Design	Type of EE	Condition	N	Age mean (SD) / % total	Composition of cost	Composition of direct cost
董晓春 2011[1]	Eastern China	2	Societal	Retrospective cohort study	COI	FLU	167	≤60y(98.20%) ≥60y(1.80%)	I	NA
田雪莹 2012[2]	Eastern China	NA	Hospital	Cross-sectional study	COI	FLU	183	0-20y(44.81%) 20-40y(32.24%) 40-60y(13.11%) ≥60y(8.20%)	D	MC
邸明芝 2014[3]	Eastern China	2，3	Hospital	Cross-sectional study	COI	CAP	248	≤60y(43.15%) ≥60y(56.85%)	D	MC
Yang J 2015[4]	NA	NA	Societal	Cross-sectional study	COI	FLU	783	0-5y(33.97%) 5-14y(39.72%) 15-59y(23.88%) ≥60y(2.43%)	D+I	MC+nMC
刘慧 2016[5]	Eastern China	NA	Hospital	Cross-sectional study	COI	ARI	48886	0-4y(39.38%) 5-14y(10.04%) 15-64y(22.29%) ≥65y(28.29%)	D	MC
马为 2007[6]	Eastern China	3	Hospital	Clinical trial	CEA	CAP	120	66.32y(11.14)	D	MC
程克文 2009[7]	Eastern China	NA	Hospital	Cross-sectional study	COI	CAP	3502	75.03y(8.42)	D	MC
程真顺 2010[8]	Central China	3	Hospital	Cross-sectional study	COI	CAP	125	75.2y(12.0)	D	MC
唐丽君 2010[9]	Eastern China	3	Hospital	Retrospective cohort study	COI	CAP	50	≤60y(44.00%) ≥60y(56.00%)	D	MC
王茂慈 2010[10]	Eastern China	3	Hospital	Prospective cohort study	CEA	FLU	339	74.96y(6.22)	D	MC
梁颖 2012[11]	Eastern China	3	Hospital	Prospective cohort study	COI	CAP	60	18-64y(41.67%) ≥65y(58.33%)	D	MC
廖慧中	Central	3	Hospital	Community	CEA	CAP	169	73.4y	D	MC

2012[12]	China			trial						
张音 2012[13]	NA	3	Hospital	Retrospective cohort study	COI	CAP	4567	≥60y(100.00%)	D	MC
黄建会 2013[14]	Western China	3	Hospital	Clinical trial	COI	CAP	40	≥60y(100.00%)	D	MC
宋圣帆 2013[15]	Eastern China	1，2，3	Hospital	Cross-sectional study	COI	CAP	27973	0-1y(7.91%) 2-4y(8.44%) 5-17y(8.21%) 18-34y(5.54%) 35-49y(6.75%) 50-64y(18.50%) ≥65y(44.65%)	D	MC
Zhou L. 2013[16]	NA	3	Hospital	Cross-sectional study	COI	CAP	106	≤15y(60.38%) 16-64y(21.70%) ≥65y(17.92%)	D	MC
杨娟 2015[17]	NA	3	Hospital	Cross-sectional study	COI	ARI	122	≤5y(55.74%) 5-14y(18.85%) 15-59y(17.21%) ≥60y(8.20%)	D+I	MC+nMC
Chen Jian 2015[18]	Eastern China	NA	Societal	Cross-sectional study	COI	FLU	1777	≥60y(100.00%)	D+I	MC+nMC
井奚月 2016[19]	Eastern China	1，2，3	Hospital	Cross-sectional study	COI	CAP	10963	68y	D	MC
尹洪珍 2016[20]	Eastern China	1	Hospital	Clinical trial	CEA	CAP	120	57.38y(6.95)	D	MC
Huo X 2016[21]	Eastern China	NA	Hospital	Cross-sectional study	COI	FLU	52	54.5y	D	MC
刘慧 2017[22]	Eastern China	NA	Hospital	Cross-sectional study	COI	ARI	14308	60-74y(39.77%) 75-89y(53.84%) ≥90y(6.38%)	D	MC
倪宁 2017[23]	Western China	3	Hospital	Clinical trial	CEA	CAP	59	58-79y(100%)	D	MC
闫建华 2017[24]	Central China	2	Hospital	Cross-sectional study	COI	CAP	244	80.73y	D	MC

闫郑伟 2017[25]	Western China	3	Hospital	Community trial	CEA	CAP	165	≥65y(100.00%)	D	MC
Li X 2019[26]	Eastern China	NA	Societal	Cross-sectional study	COI	CAP	323992	0-4y(5.65%) 5-19y(3.50%) 20-49y(5.00%) 50-64y(10.15%) 65-74y(12.06%) 75-84y(29.25%) ≥85y(34.39%)	D	MC
董莹 2020[27]	Eastern China	2，3	Hospital	Cross-sectional study	COI	CAP	52247	≤1y(18.37%) 1-6y(32.69%) 6-19y(8.83%) 19-60y(16.37%) ≥60y(23.74%)	D	MC
刘令初 2020[28]	Eastern China	NA	Societal	Prospective cohort study	CEA	FLU	2094	≥60y(100.00%)	D+I	MC+nMC
王静 2020[29]	Eastern China	2	Hospital	Clinical trial	CEA	CAP	122	≥65y(100.00%)	D	MC
Li X. Z. 2020[30]	Eastern China	NA	Hospital	Cross-sectional study	COI	COVID-19	70	52y	D	MC
涂正波 2021[31]	Central China	NA	Societal	Cross-sectional study	COI	FLU	141	0-4y(31.91%) 5-13y(39.01%) 14-59y(17.73%) ≥60y(11.35%)	D+I	MC
吴一峰 2021[32]	Eastern China	2，3	Hospital	Prospective cohort study	CEA	FLU	388	≥60y(100.00%)	D+I	MC+nMC
Lai X 2021[33]	NA	NA	Societal	Cross-sectional study	COI	FLU	12252	0-2y(30.42%) 3-5y(24.00%) 18-39y(0.59%) 40-49y(2.55%) 50-59y(11.02%) 60-69y(16.69%) 70-79y(12.17%) ≥80y(2.55%)	D+I	MC+nMC

徐英 2005[34]	Western China		Societal	Community trial	CEA	ARI	600	≥60y(100.00%)	D	MC
麦华玉 2012[35]	Eastern China	2	Hospital	Clinical trial	CEA	CAP	124	≥60y(100.00%)	D	MC
陈健 2013[36]	Eastern China		Hospital	Cross-sectional study	COI	FLU	1804	60-70y(64.97%)<br>＞70y(35.03%)	D	MC+nMC
程念 2013[37]	NA		Hospital	Cross-sectional study	COI	CAP	6599	≥60y(100.00%)	D	MC
张雅娟 2013[38]	Eastern China	1	Hospital	Cross-sectional study	COI	CAP	122	80.73y	D	MC
张晓羽 2015[39]	Eastern China	3	Hospital	Clinical trial	CEA	CAP	146	≥60y(100.00%)	D	MC
甘语波 2018[40]	Western China	2	Hospital	Retrospective cohort study	COI	CAP	84	≤60y(50%)<br>＞60y(50%)	D	MC
孙聪 2019[41]	Eastern China	3	Hospital	Cross-sectional study	COI	CAP	190	14-19y<br>20-29y<br>30-39y<br>40-49y<br>50-59y<br>60-69y<br>70-79y<br>≥80y	D	MC
尹超男 2021[42]	Central China		Societal	Cross-sectional study	COI	COVID-19	1272	0-4y(0.79%)<br>5-14y(2.28%)<br>15-29y(15.49%)<br>30-44y(29.80%)<br>45-59y(36.71%)<br>60-69y(8.65%)<br>70-79y(4.48%)<br>≥80y(1.81%)	I	MC
黄文姬 2015[43]	Eastern China	1	Hospital	Community trial	CEA	CAP	200	69.7y(6.5)	D	MC
梁燕芳 2015[44]	Eastern China	1	Hospital	Community trial	CEA	CAP	205	74y	D	MC
李朝晖 2016[45]	Northeastern China	2	Hospital	Community trial	CEA	CAP	100	≥60y(100.00%)	D	MC

那坤艳 2016[46]	Northeastern China	1	Hospital	Community trial	CEA	CAP	160	≥60y(100.00%)	D	MC
瞿玉仙 2016[47]	Eastern China	2	Hospital	Community trial	CEA	CAP	70	71.1y	D	MC
谭星宇 2002[48]	Eastern China	3	Hospital	Cross-sectional study	COI	CAP	362	＜45y(30.11%) 45-65y(29.83) ≥65y(40.06%)	D	MC
董军 2003[49]	Eastern China	3	Hospital	Cross-sectional study	COI	SARS	680	＜40y(65.44%) 40-59y(28.68%) ≥60y(5.88%）	D	MC
肖峰 2004[50]	Eastern China		Hospital	Cross-sectional study	COI	SARS	1272	＜18y(4.25%） 18-40y(59.36%) 40-65y(29.01%) ≥65y(7.39%)	D	MC
王红军 2005[51]	Western China	3	Hospital	Retrospective cohort study	COI	CAP	207	≤60y(83.09%) ＞60y(16.91%)	D	MC
王建华 2006[52]	Eastern China		Hospital	Cross-sectional study	COI	SARS	1398		D	MC
姚盛思 2006[53]	Western China	3	Hospital	Clinical trial	CEA	CAP	42	≥65y(100.00%)	D	MC
关华 2007[54]	Eastern China		Hospital	Cross-sectional study	COI	SARS	1353	0-20y(10.57%) 20-40y(53.22%) 40-60y(26.24%) ≥60y(11.46%)	D	MC
程真顺 2011[55]	Central China	3	Hospital	Prospective cohort study	COI	CAP	432	<65y(31.48%) ≥65y(68.52%)	D	MC
闫翔 2011[56]	Western China	3	Hospital	Clinical trial	CEA	CAP	42	≥60y(100.00%)	D	MC
李志杰 2012[57]	Central China	3	Hospital	Clinical trial	CEA	CAP	57	60.5y(3.5)	D	MC
陈志燕 2013[58]	Eastern China	2	Hospital	Clinical trial	CEA	CAP	120	≥60y(100.00%)	D	MC
顾建英 2013[59]	Eastern China	3	Hospital	Retrospective cohort	COI	CAP	143	<60y(32.17%) ≥60y(67.83%)	D	MC

				study						
李宏 2013[60]	Western China	3	Hospital	Clinical trial	CEA	CAP	58	≥65y(100.00%)	D	MC
李秀荣 2013[61]	Eastern China	3	Hospital	Clinical trial	CEA	CAP	120	75.4y	D	MC
张益辉 2013[62]	Eastern China	2	Hospital	Prospective cohort study	COI	CAP	111	≥50y(100.00%)	D	MC
钱玉英 2014[63]	Eastern China	3	Hospital	Clinical trial	CEA	CAP	112	≥65y(100.00%)	D	MC
王玉梅 2014[64]	Eastern China	3	Hospital	Clinical trial	CEA	CAP	282	≥65y(100.00%)	D	MC
张晓辉 2014[65]	Eastern China	2	Hospital	Clinical trial	CEA	CAP	62	75.3y(10.8)	D	MC
陈妍 2015[66]	Western China	3	Hospital	Clinical trial	CEA	CAP	100	≥60y(100.00%)	D	MC
纪颖 2015[67]	Eastern China	3	Hospital	Retrospective cohort study	COI	CAP	221	<65y(57.01%) ≥65y(42.99%)	D	MC
刘美蓉 2015[68]	Eastern China	3	Hospital	Clinical trial	CEA	CAP	160	79.3y(6.4)	D	MC
万秀英 2015[69]	Eastern China	3	Hospital	Clinical trial	CEA	CAP	80	≥60y(100.00%)	D	MC
曾小云 2016[70]	Eastern China	3	Hospital	Case-control study	COI	CAP	347	≥65y(100.00%)	D	MC
陈建新 2016[71]	Eastern China	2	Hospital	Community trial	CEA	CAP	90	73.2y	D	MC
陈丽莉 2016[72]	Eastern China	2	Hospital	Clinical trial	CEA	CAP	80	≥60y(100.00%)	D	MC
胡循贵 2016[73]	Western China	3	Hospital	Clinical trial	CEA	CAP	84	69.0y(3.5)	D	MC
贾晓利 2016[74]	Western China	3	Hospital	Case-control study	COI	CAP	235	≥65y(100.00%)	D	MC
李音 2016[75]	Eastern China	3	Hospital	Case-control study	COI	CAP	92	60-74y(32.61%) 75-89y(38.04%) ≥90y(29.35%)	D	MC
邱小松 2016[76]	Eastern China	3	Hospital	Clinical trial	CEA	CAP	15	＞50y(100.00%)	D	MC
叶敏	Western	2	Hospital	Clinical	CEA	CAP	74	＞	D	MC

2016[77]	China			trial				50y(100.00%)		
刘蕊 2017[78]	Northeastern China	3	Hospital	Clinical trial	CEA	CAP	104	＞ 50y(100.00%)	D	MC
徐玲 2017[79]	Central China	3	Hospital	Retrospective cohort study	COI	CAP	187	≥60y(100.00%)	D	MC
李伟涛 2018[80]	Eastern China	2	Hospital	Clinical trial	CEA	CAP	200	65.90y(6.52)	D	MC
丘韶校 2018[81]	Eastern China	3	Hospital	Clinical trial	CEA	CAP	80	70.6y(10.5)	D	MC
张玉明 2018[82]	Western China	3	Hospital	Clinical trial	CEA	CAP	64	≥60y(100.00%)	D	MC
朱爱娥 2018[83]	Western China	2	Hospital	Clinical trial	CEA	CAP	90	≥60y(100.00%)	D	MC
陈英 2019[84]	Western China	3	Hospital	Cross-sectional study	COI	CAP	6163	＜3 月(23.14%） 3 月-1y(14.68%) 1-5y(20.28%) 6-64y(23.53%) ≥65y(18.37%)	D	MC
郭玉梅 2019[85]	Northeastern China	3	Hospital	Clinical trial	CEA	CAP	98	≥65y(100.00%)	D	MC
邱红侠 2019[86]	Northeastern China	1	Hospital	Community trial	CEA	CAP	106	≥60y(100.00%)	D	MC
吴双胜 2019[87]	Eastern China		Hospital	Cross-sectional study	COI	FLU	7327	18-59y(80.24%) ≥60y(19.75%)	D+I	MC+nMC
尹慧洁 2019[88]	Eastern China	3	Hospital	Clinical trial	CEA	CAP	40	＞ 50y(100.00%)	D	MC
李义平 2020[89]	Eastern China		Hospital	Cross-sectional study	COI	COVID-19	105	≤18y(12.38%) 19-40y(40.95%) 41-64y(36.19%) ≥65y(10.48%)	D	MC
吴学智 2020[90]	Western China	3	Hospital	Cross-sectional study	COI	CAP	3814	≤44y(48,87%) 45-59y(16.94%) ≥60y(34.19%)	D	MC
肖祖华 2020[91]	Eastern China	2	Hospital	Clinical trial	CEA	CAP	86	≥60y(100.00%)	D	MC

刘杰 2021[92]	Eastern China	3	Hospital	Cross-sectional study	COI	CAP	7462		D	MC
涂正波 2021[93]	Central China		Hospital	Cross-sectional study	COI	FLU	682	0-4y(21.70%) 5-9y(46.04%) 10-14y(14.96%) 15-19y(3.37%) 20-24y(2.49%) 25-29y(1.76%) 30-34y(1.61%) 35-39y(0.88%) 40-44y(0.88%) 45-49y(0.44%) 50-54y(0.15%) 55-59y(0.73%) ≥60y(4.99%)	D+I	MC
陈艳艳 2022[94]	Central China	2	Hospital	Clinical trial	CEA	CAP	70	＞60y(100.00%)	D	MC
胡婷婷 2022[95]	Western China		Hospital	Cross-sectional study	COI	COVID-19	457	0-17y(6.56) 18-45y(55.80) 46-64y(30.85) ≥65y(6.78)	D	MC
李燕菊 2022[96]	Western China	3	Hospital	Cross-sectional study	COI	CAP	1211	≥65y(100.00%)	D	MC
李育梅 2022[97]	Eastern China	3	Hospital	Cross-sectional study	COI	COVID-19	96	＜40y(14.58) 40-59y(19.79) ≥60y(8.33)	D	MC
任秀君 2022[98]	Western China	3	Hospital	Clinical trial	CEA	CAP	120	≥60y(100.00%)	D	MC
徐江奇 2022[99]	Central China	3	Hospital	Case-control study	COI	CAP	120	≥60y(100.00%)	D	MC

EE: economic evaluation; SD: standard deviation; NA: not available; COI: cost-of-illness analysis; CEA: cost-effectiveness analysis; FLU: influenza; CAP: community acquired pneumonia; ARI: acute respiratory disease; COVID-19: corona virus disease 2019; SARS: severe acute respiratory syndrome; I: indirect cost; D: direct cost; MC: medical cost; nMC: non-medical cost.

Table S5. Quality and Potential for Bias

Author Year	Q1	Q2	Q3	Q4	Q5	Q6	Q7	Q8	Q9	Q10	Q11	Q12	Q13	Q14	Q15	Total Score
董晓春 2011[1]	YES	YES	YES	YES	YES	YES	NA	YES	NO	NA	YES	YES	YES	YES	YES	12
田雪莹 2012[2]	YES	YES	YES	YES	NO	YES	NA	YES	NO	NA	YES	YES	YES	YES	YES	11
邸明芝 2014[3]	YES	YES	NO	YES	NO	NO	NA	YES	NO	NA	YES	YES	YES	YES	YES	9
Yang J 2015[4]	YES	YES	YES	YES	YES	YES	NA	YES	YES	NA	YES	YES	YES	YES	YES	13
刘慧 2016[5]	YES	YES	YES	YES	NO	YES	NA	YES	NO	NA	YES	YES	NO	YES	YES	10
马为 2007[6]	YES	YES	YES	YES	YES	YES	NA	YES	YES	YES	YES	YES	NO	NO	NO	11
程克文 2009[7]	YES	NO	YES	YES	NO	YES	NA	YES	NO	NA	YES	YES	YES	NO	YES	9
程真顺 2010[8]	YES	NO	YES	YES	NO	YES	NA	NO	NO	NO	YES	YES	NO	NO	YES	7
唐丽君 2010[9]	YES	NO	YES	YES	NO	YES	NA	YES	NO	NA	YES	YES	NO	NO	YES	8
王茂慈 2010[10]	YES	YES	YES	YES	NO	YES	NA	NO	NO	YES	YES	YES	NO	YES	YES	10
梁颖 2012[11]	YES	NO	YES	YES	NO	YES	NA	YES	NO	NA	YES	YES	NO	NO	YES	8
廖慧中 2012[12]	YES	NO	YES	YES	NO	YES	NA	YES	NO	YES	YES	YES	NO	NO	YES	9
张音 2012[13]	YES	YES	YES	NO	NO	YES	NA	YES	NO	NA	YES	YES	NO	YES	YES	9
黄建会 2013[14]	YES	NO	YES	YES	NO	YES	NA	YES	NO	YES	YES	YES	NO	NO	YES	9
宋圣帆 2013[15]	YES	YES	YES	YES	YES	YES	YES	YES	NO	NO	YES	YES	YES	YES	YES	13
Zhou L.2013[16]	YES	YES	YES	NO	YES	YES	NA	YES	NO	NA	YES	YES	YES	YES	YES	11
杨娟 2015[17]	YES	YES	NO	YES	YES	NO	NA	YES	YES	NA	YES	YES	YES	YES	YES	11
Chen Jian2015[18]	YES	YES	YES	YES	YES	YES	NA	YES	NO	NA	YES	YES	YES	YES	YES	12
井奚月 2016[19]	YES	YES	YES	YES	YES	YES	NA	YES	YES	NA	YES	YES	YES	NO	YES	12
尹洪珍 2016[20]	YES	YES	YES	YES	NO	YES	NA	YES	NO	YES	YES	YES	NO	NO	YES	10
Huo X2016[21]	YES	YES	NO	YES	NO	NO	NA	YES	NO	NA	YES	YES	YES	YES	YES	9
刘慧 2017[22]	YES	YES	YES	YES	YES	YES	YES	YES	NO	NA	YES	YES	NO	YES	YES	12

倪宁 2017[23]	YES	YES	YES	YES	NO	YES	NA	YES	NO	YES	YES	YES	NO	NO	YES	10
闫建华 2017[24]	YES	NO	YES	YES	NO	YES	NA	NO	NO	NA	YES	YES	NO	NO	NO	6
闫郑伟 2017[25]	YES	YES	YES	YES	NO	YES	NA	YES	NO	YES	YES	YES	NO	NO	YES	10
Li X2019[26]	YES	YES	YES	YES	YES	YES	NA	YES	NO	NA	YES	YES	YES	YES	YES	12
董莹 2020[27]	YES	YES	YES	YES	NO	YES	NA	YES	NO	NA	YES	YES	NO	NO	YES	9
刘令初 2020[28]	YES	YES	NO	YES	NO	NO	NA	YES	NO	YES	YES	YES	YES	YES	YES	10
王静 2020[29]	YES	YES	YES	YES	NO	YES	NA	YES	NO	YES	YES	YES	NO	NO	YES	10
Li X. Z.2020[30]	YES	YES	NO	YES	YES	NO	NA	YES	NO	NA	YES	YES	YES	YES	YES	10
涂正波 2021[31]	YES	YES	NO	YES	NO	NO	NO	YES	NO	NA	YES	YES	NO	YES	YES	8
吴一峰 2021[32]	YES	YES	YES	YES	NO	YES	NA	YES	NO	YES	YES	YES	YES	YES	YES	12
Lai X2021[33]	YES	YES	NO	YES	NO	NO	NA	YES	NO	NA	YES	YES	YES	YES	YES	9
徐英 2005[34]	YES	YES	YES	NO	NO	NO	NA	YES	NO	YES	NO	YES	NO	YES	YES	8
麦华玉 2012[35]	YES	YES	YES	YES	NO	YES	NA	YES	NO	YES	NO	YES	NO	NO	YES	9
陈健 2013[36]	YES	YES	YES	YES	YES	YES	NA	YES	NO	NA	YES	YES	NO	YES	YES	11
程念 2013[37]	YES	YES	YES	YES	YES	YES	NA	NO	NO	NA	YES	YES	NO	NO	YES	9
张雅娟 2013[38]	YES	NO	YES	YES	YES	YES	NA	NO	NO	NA	YES	YES	NO	NO	YES	8
张晓羽 2015[39]	YES	YES	YES	YES	NO	YES	NA	YES	NO	YES	YES	YES	NO	NO	YES	10
甘语波 2018[40]	YES	NO	YES	YES	YES	YES	NA	YES	NO	NA	NO	YES	NO	NO	YES	8
孙聪 2019[41]	YES	NO	YES	YES	YES	YES	NA	YES	NO	NA	YES	YES	YES	YES	YES	11
尹超男 2021[42]	YES	YES	NO	YES	NO	NO	NA	YES	NO	NA	YES	YES	YES	NO	YES	8
黄文姬 2015[43]	YES	YES	YES	YES	NO	YES	NA	YES	NO	YES	NO	YES	NO	NO	YES	9
梁燕芳 2015[44]	YES	YES	YES	YES	YES	YES	NA	YES	NO	YES	YES	YES	NO	NO	YES	11
李朝晖 2016[45]	YES	YES	YES	YES	YES	YES	NA	YES	NO	YES	YES	YES	NO	NO	YES	11
那坤艳 2016[46]	YES	YES	YES	YES	NO	YES	NA	YES	NO	YES	YES	YES	NO	NO	YES	10
瞿玉仙 2016[47]	YES	YES	NO	NO	NO	NO	NA	YES	NO	YES	NO	YES	NO	NO	YES	6
谭星宇 2002[48]	YES	YES	NO	YES	YES	NO	NA	YES	NO	NA	YES	YES	NO	NO	YES	8

董军 2003[49]	YES	YES	YES	YES	NO	YES	NA	NO	NO	NA	YES	YES	NO	YES	YES	9
肖峰 2004[50]	YES	YES	YES	YES	YES	YES	NA	YES	NO	NA	YES	YES	NO	YES	YES	11
王红军 2005[51]	YES	NO	YES	YES	NO	YES	NA	YES	NO	NA	YES	YES	NO	NO	YES	8
王建华 2006[52]	YES	YES	YES	YES	YES	YES	NA	YES	NO	NA	YES	YES	NO	YES	YES	11
姚盛思 2006[53]	YES	YES	YES	YES	NO	YES	NA	NO	NO	YES	NO	YES	NO	NO	YES	8
关华 2007[54]	YES	YES	YES	YES	NO	YES	NA	YES	NO	NA	YES	YES	NO	YES	YES	10
程真顺 2011[55]	YES	NO	YES	YES	NO	YES	NA	YES	NO	NA	YES	YES	NO	YES	YES	9
闫翔 2011[56]	YES	YES	YES	YES	NO	YES	NA	YES	NO	YES	YES	YES	NO	NO	YES	10
李志杰 2012[57]	YES	YES	YES	YES	NO	YES	NA	YES	NO	YES	NO	YES	NO	NO	YES	9
陈志燕 2013[58]	YES	YES	YES	YES	NO	YES	NA	YES	NO	YES	YES	YES	NO	NO	YES	10
顾建英 2013[59]	YES	NO	YES	YES	NO	YES	NA	YES	NO	NA	YES	YES	NO	YES	YES	9
李宏 2013[60]	YES	YES	YES	YES	NO	YES	NA	YES	NO	YES	YES	YES	NO	NO	YES	10
李秀荣 2013[61]	YES	YES	YES	YES	NO	YES	NA	YES	NO	YES	YES	YES	NO	NO	YES	10
张益辉 2013[62]	YES	YES	YES	YES	NO	YES	NA	YES	NO	NO	YES	YES	NO	NO	YES	9
钱玉英 2014[63]	YES	YES	YES	YES	NO	YES	NA	YES	NO	YES	NO	YES	NO	YES	YES	10
王玉梅 2014[64]	YES	YES	YES	YES	NO	YES	NA	YES	NO	YES	NO	YES	NO	NO	YES	9
张晓辉 2014[65]	YES	YES	YES	YES	NO	YES	NA	YES	NO	YES	YES	YES	NO	YES	YES	11
陈妍 2015[66]	YES	YES	YES	YES	NO	YES	NA	YES	NO	YES	NO	YES	NO	NO	YES	9
纪颖 2015[67]	YES	NO	YES	YES	NO	YES	NA	YES	NO	NA	YES	YES	NO	NO	YES	8
刘美蓉 2015[68]	YES	YES	YES	YES	NO	YES	NA	YES	NO	YES	YES	YES	NO	NO	YES	10
万秀英 2015[69]	YES	YES	YES	YES	NO	YES	NA	YES	NO	YES	YES	YES	NO	NO	YES	10
曾小云 2016[70]	YES	NO	YES	YES	NO	YES	NA	YES	NO	NA	YES	YES	YES	NO	YES	9
陈建新 2016[71]	YES	YES	YES	NO	YES	YES	NA	YES	NO	YES	NO	YES	NO	NO	YES	9
陈丽莉 2016[72]	YES	YES	YES	YES	NO	YES	NA	YES	NO	YES	NO	YES	NO	NO	YES	9
胡循贵 2016[73]	YES	YES	YES	YES	NO	YES	NA	YES	NO	YES	YES	YES	NO	NO	YES	10
贾晓利 2016[74]	YES	NO	YES	YES	NO	YES	NA	YES	NO	NA	YES	YES	NO	NO	YES	8

李音 2016[75]	YES	NO	YES	YES	NO	YES	NA	YES	NO	NA	YES	YES	NO	NO	YES	8
邱小松 2016[76]	YES	YES	YES	YES	NO	YES	NA	YES	NO	YES	YES	YES	NO	NO	YES	10
叶敏 2016[77]	YES	YES	YES	YES	NO	YES	NA	YES	NO	YES	YES	YES	NO	NO	YES	10
刘蕊 2017[78]	YES	YES	YES	YES	NO	YES	NA	YES	NO	YES	YES	YES	NO	NO	YES	10
徐玲 2017[79]	YES	NO	YES	YES	NO	YES	NA	YES	NO	NA	YES	YES	NO	NO	YES	8
李伟涛 2018[80]	YES	YES	YES	NO	NO	YES	NA	YES	NO	YES	YES	YES	NO	NO	YES	9
丘韶校 2018[81]	YES	YES	YES	YES	NO	YES	NA	YES	NO	YES	YES	YES	NO	NO	YES	10
张玉明 2018[82]	YES	YES	YES	YES	NO	YES	NA	YES	NO	YES	NO	YES	NO	NO	YES	9
朱爱娥 2018[83]	YES	YES	YES	YES	NO	YES	NA	YES	NO	YES	YES	YES	NO	NO	YES	10
陈英 2019[84]	YES	YES	YES	YES	NO	YES	NA	YES	NO	NA	NO	YES	NO	YES	YES	9
郭玉梅 2019[85]	YES	YES	YES	YES	NO	YES	NA	YES	NO	YES	YES	YES	NO	NO	YES	10
邱红侠 2019[86]	YES	YES	YES	YES	NO	YES	NA	YES	NO	YES	NO	YES	NO	NO	YES	9
吴双胜 2019[87]	YES	YES	YES	YES	NO	NO	NA	YES	NO	NA	NO	YES	YES	YES	YES	9
尹慧洁 2019[88]	YES	YES	YES	YES	NO	YES	NA	YES	NO	YES	NO	YES	NO	NO	YES	9
李义平 2020[89]	YES	YES	YES	YES	YES	YES	NA	YES	NO	NA	YES	YES	YES	YES	YES	12
吴学智 2020[90]	YES	YES	YES	NO	NO	NO	NA	YES	NO	NA	YES	YES	NO	NO	YES	7
肖祖华 2020[91]	YES	YES	YES	YES	NO	YES	NA	YES	NO	YES	YES	YES	NO	NO	YES	10
刘杰 2021[92]	YES	YES	YES	NO	YES	YES	NA	YES	NO	NA	YES	YES	YES	YES	YES	11
涂正波 2021[93]	YES	YES	NO	YES	NO	NO	NA	YES	NO	NA	YES	YES	YES	YES	YES	9
陈艳艳 2022[94]	YES	YES	YES	YES	NO	YES	NA	YES	NO	YES	NO	YES	NO	NO	YES	9
胡婷婷 2022[95]	YES	YES	YES	NO	YES	YES	NA	YES	NO	NA	YES	YES	YES	NO	YES	10
李燕菊 2022[96]	YES	NO	NO	YES	YES	NO	NA	YES	NO	NA	YES	YES	NO	YES	YES	8
李育梅 2022[97]	YES	YES	YES	NO	YES	YES	NA	YES	NO	NA	YES	YES	NO	YES	YES	10
任秀君 2022[98]	YES	YES	YES	YES	NO	YES	NA	YES	NO	YES	YES	YES	NO	YES	YES	11
徐江奇 2022[99]	YES	NO	YES	YES	NO	YES	NA	YES	NO	NA	YES	YES	NO	NO	YES	8

See Table S 3 for details of all fifteen questions above.

Table S6. Sensitivity analysis 1

Summary of median (IQR) direct medical cost per episode of ARI in USD of 2021 among studies with quality scores of ≥11 points

Age Group	Inpatient	Outpatient
60-69	2034 (1567, 2552) (4 studies)	72 (69, 76) (2 studies)
70-79	2197 (1920, 2474) (2 studies)	–
80-	3493 (2608, 4378) (2 studies)	–
50-	2516 (1 study)	–
60-	2554 (1650, 2957) (11 studies)	73 (54, 77) (2 studies)
65-	2733 (2657, 2810) (2 studies)	–
70-	2829 (1 study)	82 (80, 85) (2 studies)

ARI: acute respiratory infection; IQR: interquartile range.

Table S7. Sensitivity analysis 2
Summary of median (IQR) direct medical cost per episode of ARI in USD of 2021 among all studies regardless of quality scores

Age Group	Inpatient	Outpatient
60-69	2669 (1833, 2991) (6 studies)	72 (69, 76) (2 studies)
70-79	2197 (1920, 2474) (2 studies)	–
80-	3493 (2608, 4378) (2 studies)	–
50-	1517 (1268, 2516) (5 studies)	–
60-	1883 (1007, 2744) (45 studies)	55 (38, 70) (6 studies)
65-	2580 (2149, 3311) (25 studies)	–
70-	1983 (1920, 2406) (3 studies)	82 (80, 85) (2 studies)

ARI: acute respiratory infection; IQR: interquartile range.

Table S8. Summary of weighted mean direct medical cost per episode of ARI in USD of 2021

Age Group	Inpatient	Outpatient
60-69	1635	72
70-79	1643	–
80-	1723	–
50-	2508	–
60-	2128	33
65-	2491	–
70-	2829	81

IQR: interquartile range; ARI: acute respiratory infection; USD: United States dollar.

Table S9. Summary of Summary of median (IQR) Length of Stay (LoS) per episode of ARI

Age Group	LoS
60-69	18 (13, 21)
70-79	12 (12, 12)
80-	14 (14, 14)
50-	15 (15, 15)
60-	18 (15, 19)
65-	18 (16, 23)
70-	20 (20, 20)

IQR: interquartile range; ARI: acute respiratory infection.

Figure S1. Geographical distribution of included studies by province

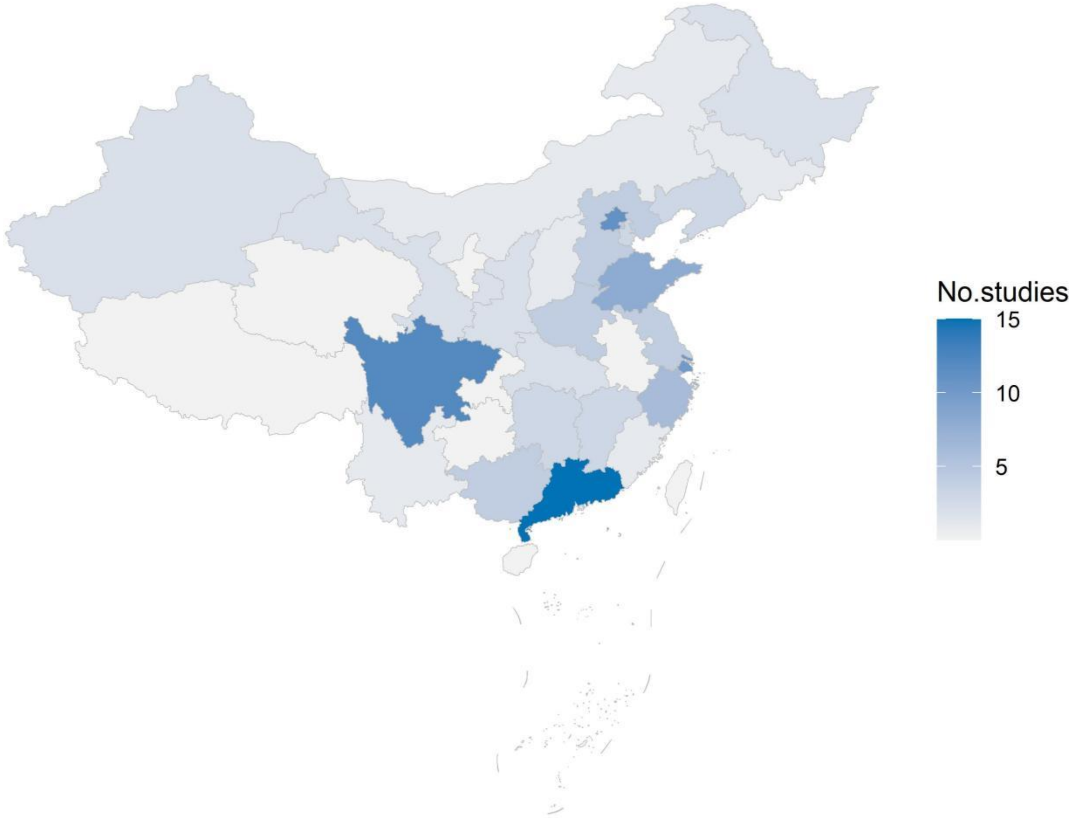

Figure S2. Histogram of the distribution of original study quality scores

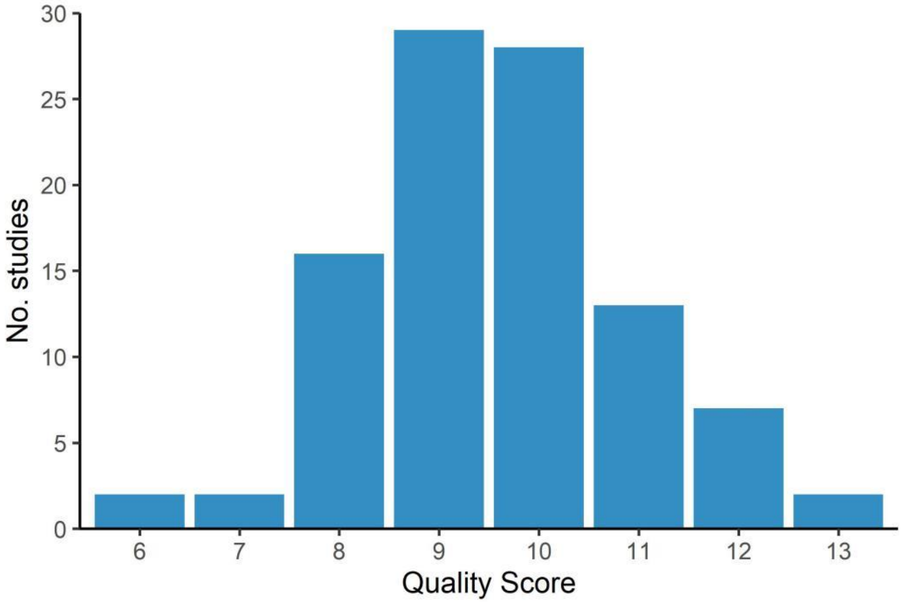

Figure S3. Correlation of results from median and weighted mean

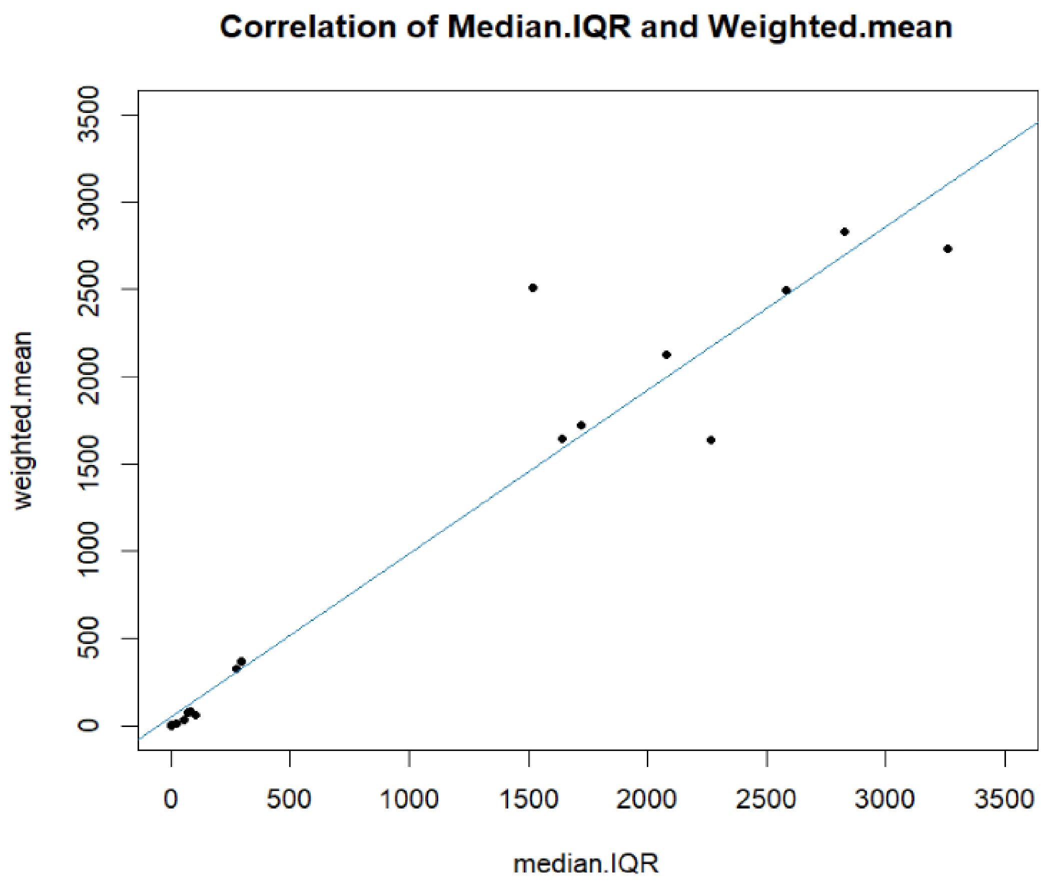

The black dots represent the median (horizontal) and weighted mean (vertical) of the cost for a specific category (age group, cost category). The blue line represents the linear correlation between the two methods. Linear correlation coefficient r = 0.966.

Figure S4. Forest plot of direct medical cost per ARI episode of inpatients aged 60 or more

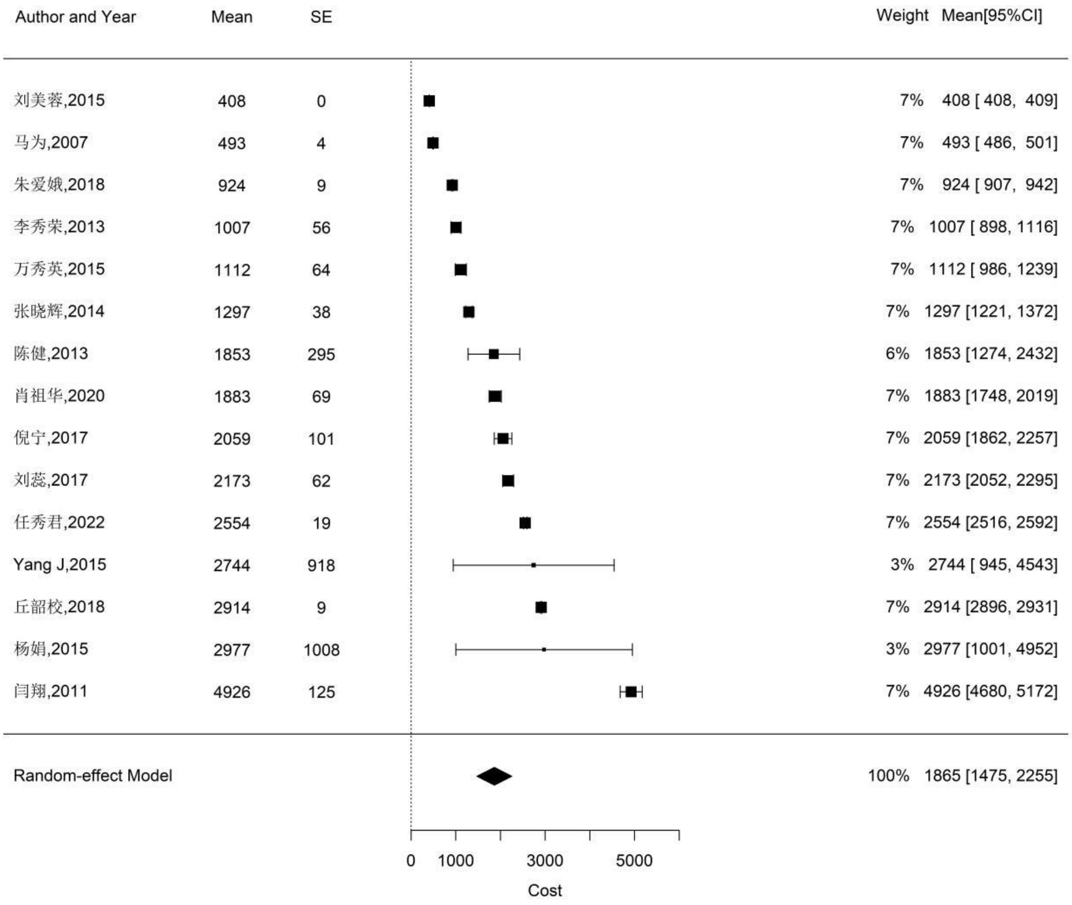

ARI: acute respiratory infection; SE: standard error; CI: confidence interval.

Figure S5. Forest plot of direct medical cost per ARI episode of inpatients aged 65 or more

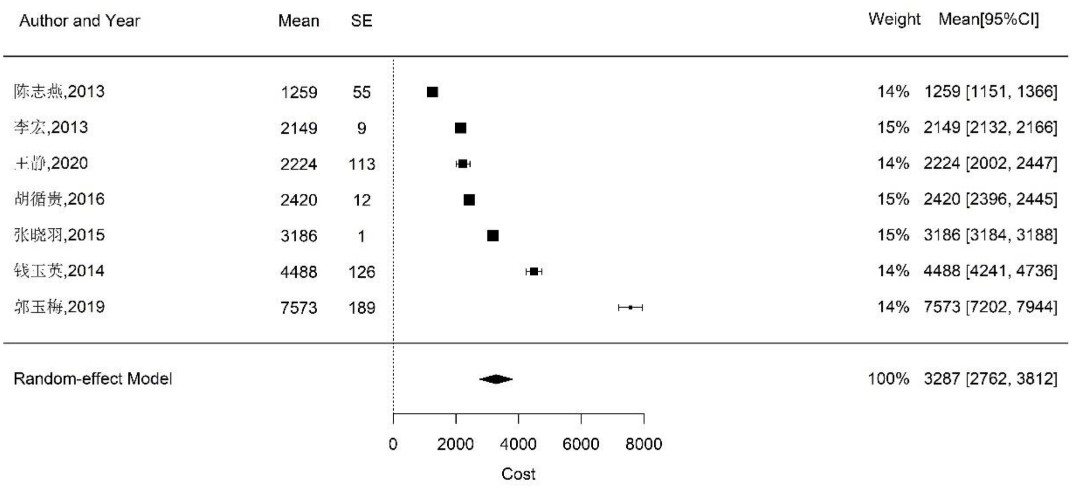

ARI: acute respiratory infection; SE: standard error; CI: confidence interval.

Figure S6. Forest plot of direct medical cost per ARI episode of inpatients of hospital grade II

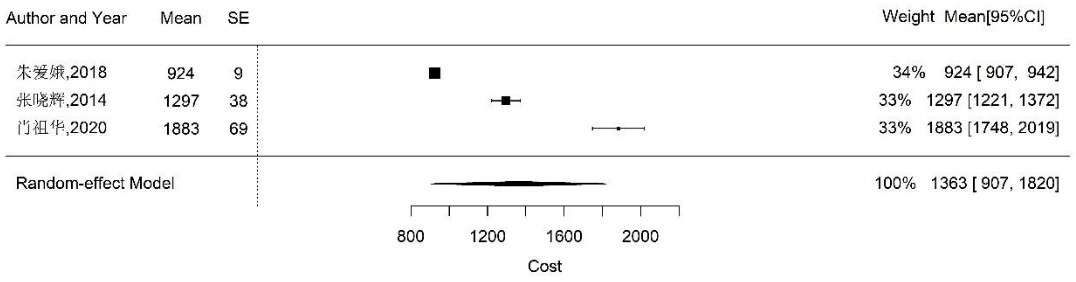

ARI: acute respiratory infection; SE: standard error; CI: confidence interval.

Figure S7. Forest plot of direct medical cost per ARI episode of inpatients of hospital grade III

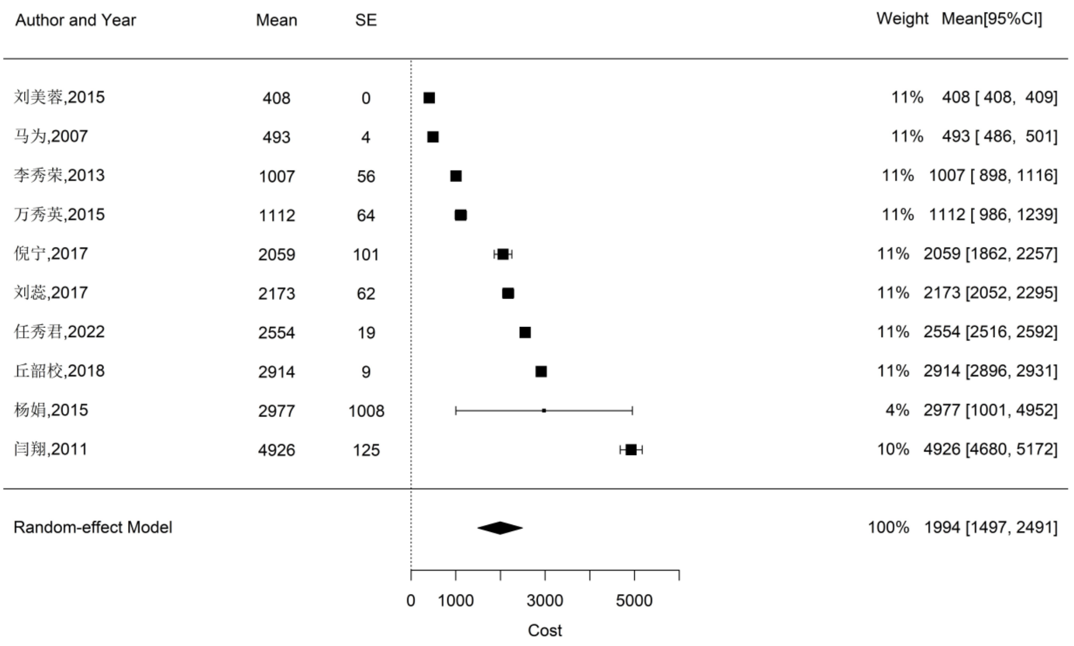

ARI: acute respiratory infection; SE: standard error; CI: confidence interval.

Figure S8. Forest plot of direct medical cost per ARI episode of inpatients of pneumonia

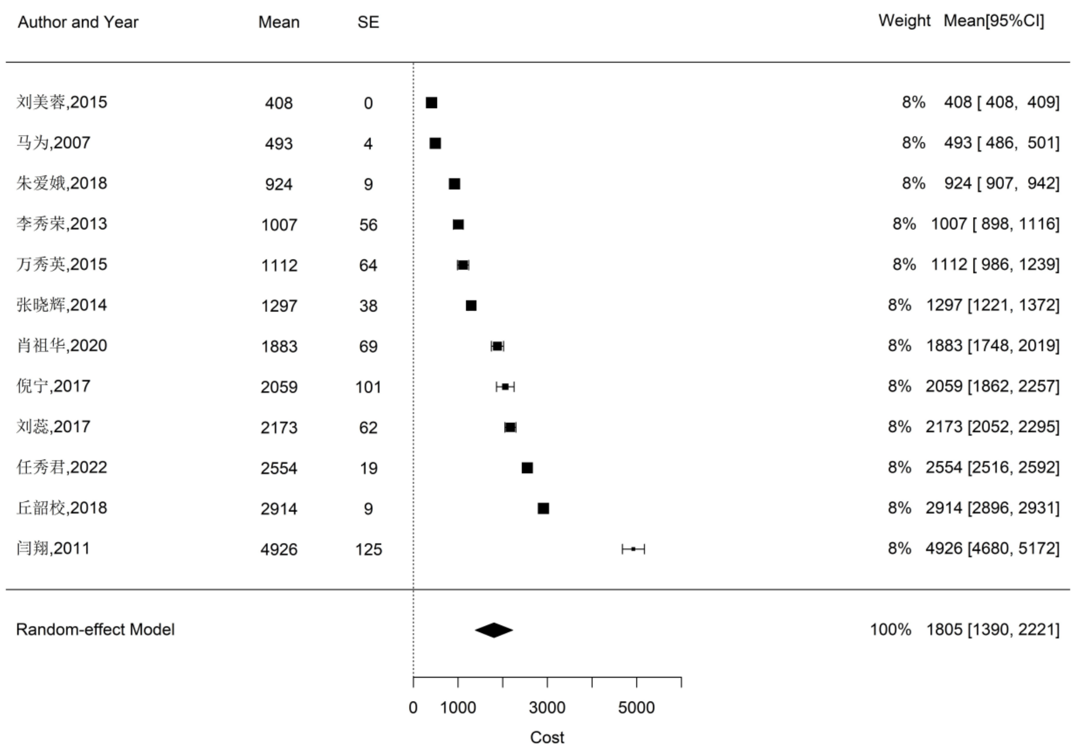

ARI: acute respiratory infection; SE: standard error; CI: confidence interval.

Figure S9. Forest plot of direct medical cost per ARI episode of inpatients of eastern China

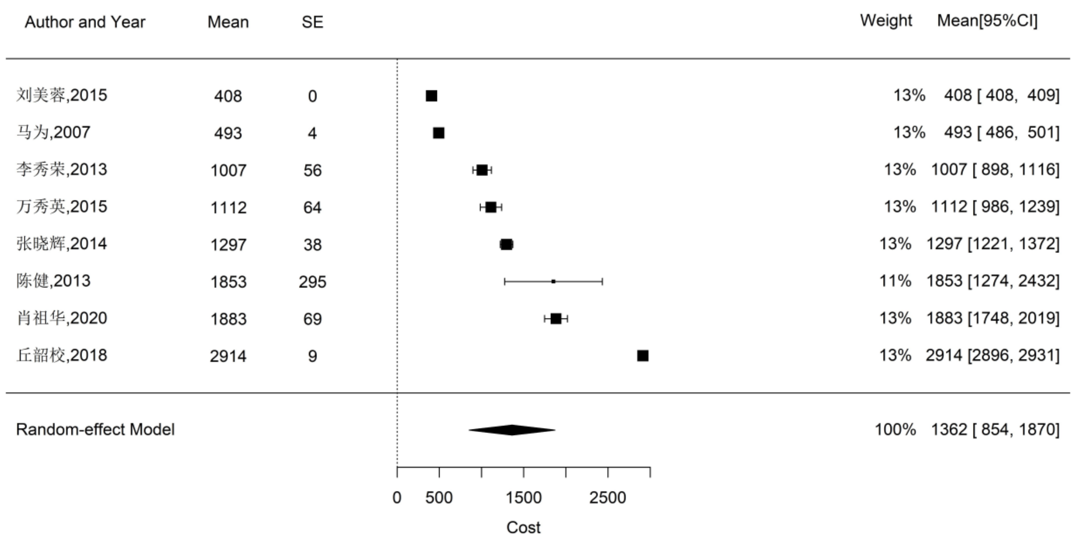

ARI: acute respiratory infection; SE: standard error; CI: confidence interval.

Figure S10. Forest plot of direct medical cost per ARI episode of inpatients of western China

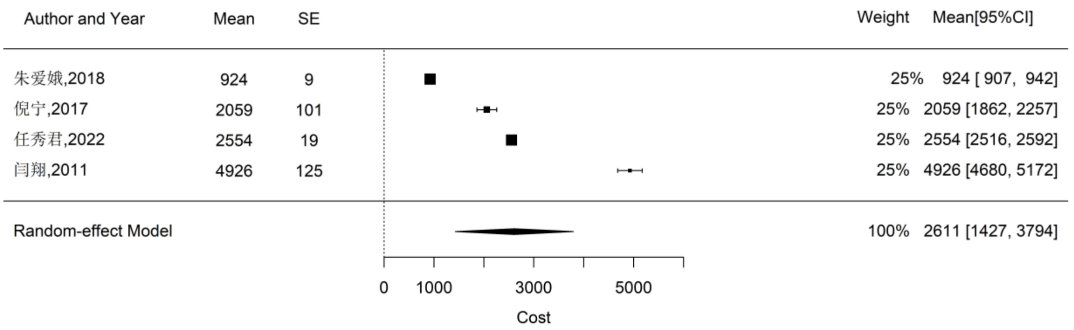

ARI: acute respiratory infection; SE: standard error; CI: confidence interval.

Figure S11. Forest plot of direct medical cost per ARI episode of inpatients of study year before 2009

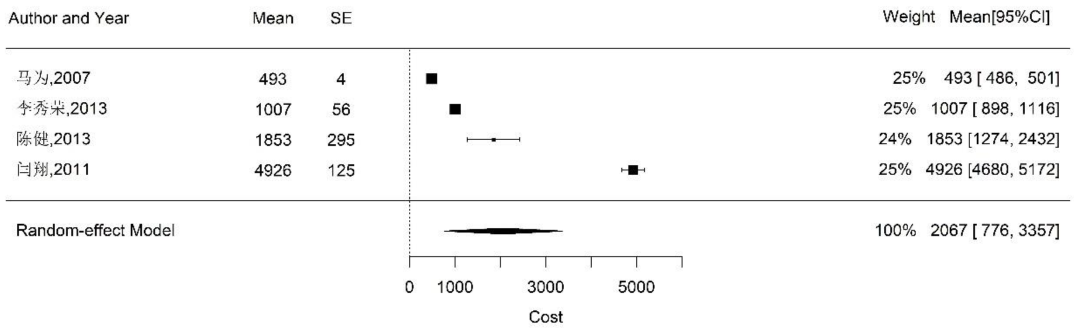

ARI: acute respiratory infection; SE: standard error; CI: confidence interval.

Figure S12. Forest plot of direct medical cost per ARI episode of inpatients of study year 2010-2014

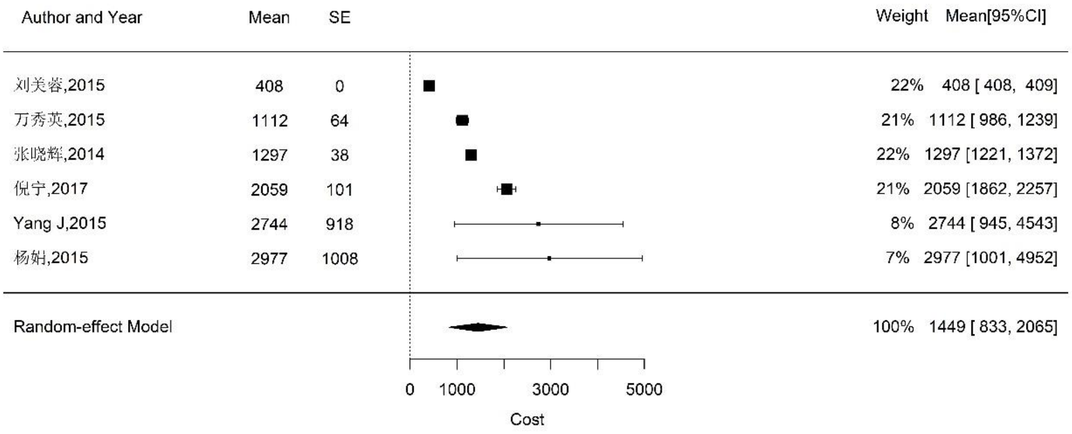

ARI: acute respiratory infection; SE: standard error; CI: confidence interval.

Figure S13. Forest plot of direct medical cost per ARI episode of inpatients of study year after 2015

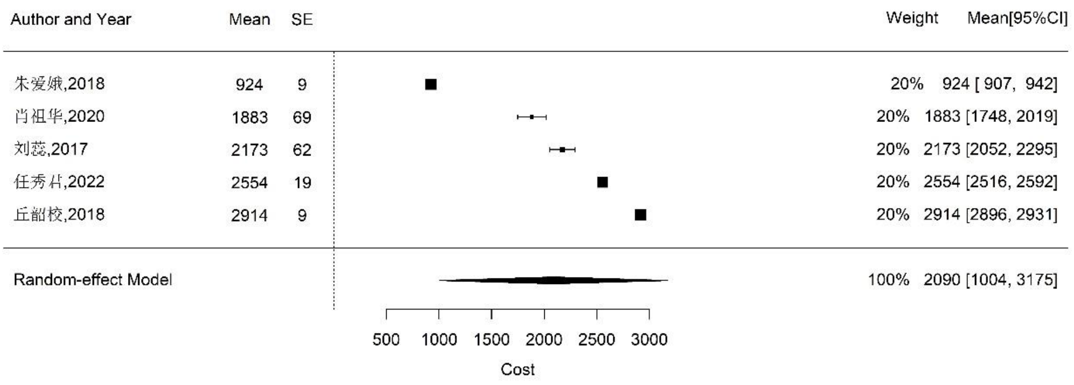

ARI: acute respiratory infection; SE: standard error; CI: confidence interval.

Figure S14. Forest plot of direct medical cost per ARI episode of inpatients of critical patients only

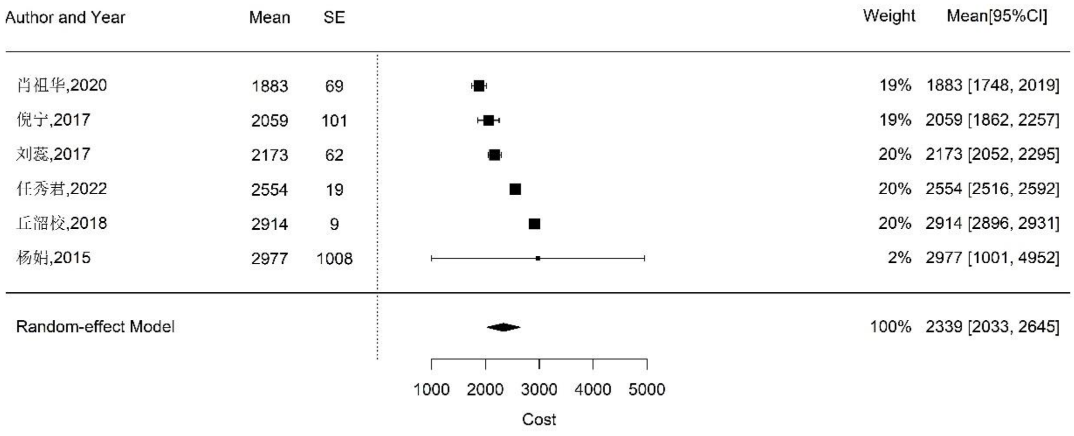

ARI: acute respiratory infection; SE: standard error; CI: confidence interval.

Figure S15. Forest plot of direct medical cost per ARI episode of inpatients of critical and noncritical patients

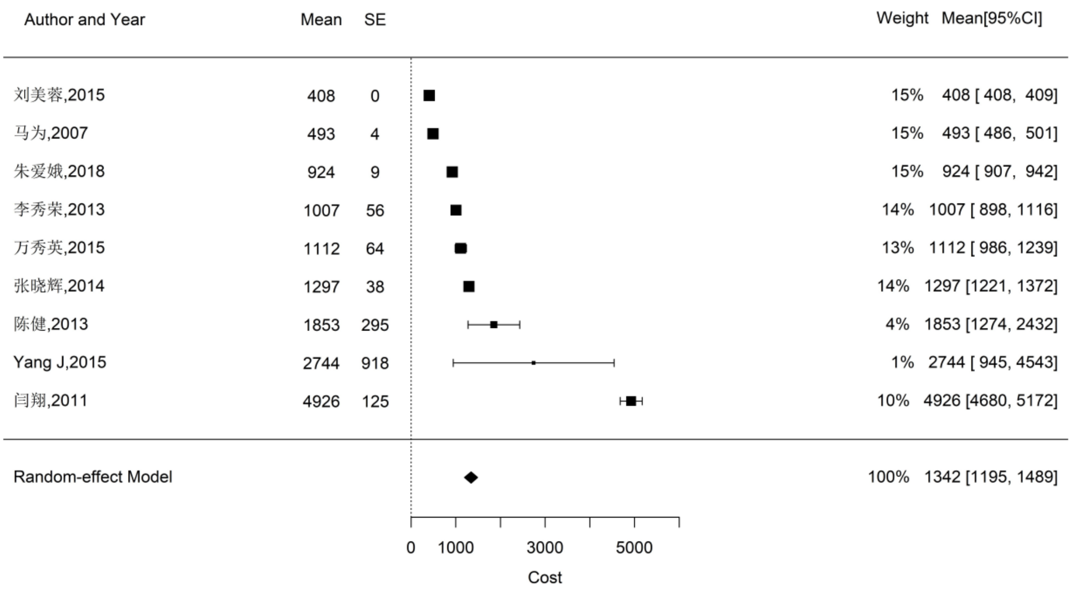

ARI: acute respiratory infection; SE: standard error; CI: confidence interval.

Supplementary Material References

1. 董晓春, 徐文体, 李琳, 张颖, 张之伦. 基于天津市汉沽街社区流行性感冒监测结果的疾病负担研究. *疾病监测*. 2011;26(10):785-787.
2. 田雪莹, 王显军, 张圣洋, 孙林, 李忠, 毕振强. 183 例甲型 H1N1 流感确诊病例住院费用及影响因素分析. *中国卫生经济*. 2012;31(07):34-36.
3. 邸明芝, 曹迎, 黄辉, et al. 248 例成人社区获得性肺炎病例疾病负担调查. *现代预防医学*. 2014;41(14):2560-2562+2584.
4. Yang J, Jit M, Leung KS, et al. The economic burden of influenza-associated outpatient visits and hospitalizations in China: a retrospective survey. *Infectious Diseases of Poverty*. Oct 6 2015;444. doi:10.1186/s40249-015-0077-6
5. 刘慧, 肖新才, 张丹萍, 陈宗遒, 陆剑云, 杨智聪. 广州市 2008—2014 年急性呼吸道感染住院病例流行特征和经济负担研究. *华南预防医学*. 2016;42(05):401-406. doi:10.13217/j.scjpm.2016.0401
6. 马为, 窦增新. 中西医治疗老年人肺炎治疗方案的成本——效果分析. *医药论坛杂志*. 2007;28(3):4-5,8. doi:10.3969/j.issn.1672-3422.2007.03.002
7. 程克文, 马佳韵, 李明珠, 张有志, 周丽, 付明明. 老年人社区获得性肺炎临床分析. *中国临床医学*. 2009;16(05):722-724.
8. 程真顺, 杨炯, 林宇辉, 叶燕青, 杨亦斌. 老年吸入性肺炎治疗及预后分析. *武汉大学学报（医学版）*. 2010;31(4):544-547.
9. 唐丽君, 葛勤敏. 社区获得性肺炎 50 例临床分析. *中国全科医学*. 2010;13(20):2271-2273. doi:10.3969/j.issn.1007-9572.2010.20.032
10. 杨晓琳 王 A 王 A 杨 A 潘 A 张 A 宋 A 殷 A. 23 价肺炎球菌多糖疫苗和流行性感冒病毒裂解疫苗联合接种对老年人呼吸系统疾病防治效果观察与效益分析. *中国疫苗和免疫*. 2010;(3)
11. 梁颖, 米玉红, 刘双. 老年与非老年患者社区获得性肺炎的临床对照研究. *中华医院感染学杂志*. 2012;22(17):3725-3727.
12. 廖慧中, 贺兼斌, 张洁, 姚娟. 综合社区护理及家庭护理干预对老年吸入综合征的预防作用. *护士进修杂志*. 2012;27(14):1309-1312. doi:10.16821/j.cnki.hsjx.2012.14.027
13. 张音, 胡剑超, 刘璟, 张步振, 刘辉. 不同付费方式下老年病人住院费用分析:以肺部感染为例. Analysis of the Cost of Hospitalization of Elderly Patients under Different Payment Methods: Taking Pulmonary Infection for Example. *中国卫生质量管理*. 2012;19(3)2012620140.
14. 黄建会, 张志鸿, 黄远琼, 黄健兰. 抗生素降阶梯治疗在重症肺炎中的疗效分析. *检验医学与临床*. 2013;(15):1990-1992. doi:10.3969/j.issn.1672-9455.2013.15.036
15. 宋圣帆. *肺炎链球菌疾病费用研究与七价肺炎球菌结合疫苗的卫生经济学评价*. 硕士. 复旦大学; 2013.
16. Zhou L, Situ S, Huang T, et al. Direct medical cost of influenza-related hospitalizations among severe acute respiratory infections cases in three provinces in China. Research Support, Non-U.S. Gov't. *PLoS ONE [Electronic Resource]*. 2013;8(5):e63788.
17. 杨娟, 郑亚明, 刘欣欣, 冯录召, 余宏杰. 中国哨点监测住院严重急性呼吸道感染病例经济负担分析. Analysis on the economic burden of severe acute respiratory infection inpatients from sentinel hospitals in China. *国际病毒学杂志*. 2015;(6)2016211144.
18. Chen J, Li Y-t, Gu B-k, Yuan Z-a. Estimation of the Direct Cost of Treating People Aged More Than 60 Years Infected by Influenza Virus in Shanghai. *Asia-Pacific Journal of Public Health*. Mar 2015;27(2):NP936-NP946. doi:10.1177/1010539512460269

19. 井奚月. *天津市参保肺炎患者住院费用分析及病例组合研究*. 硕士. 天津医科大学; 2016.
20. 尹洪珍. 血清降钙素原在老年急性社区获得性肺炎抗生素使用中的指导意义. *中国医药指南*. 2016;14(13)
21. Huo X, Chen LL, Hong L, et al. Economic burden and its associated factors of hospitalized patients infected with A (H7N9) virus: a retrospective study in Eastern China, 2013-2014. *Infectious Diseases of Poverty*. 2016;5(1):79.
22. 刘慧, 肖新才, 张丹萍, 陈宗遒, 陆剑云, 杨智聪. 2008-2014 年广州地区 8 所大型综合性医院老年人群急性呼吸道感染住院状况与直接经济负担分析. *现代预防医学*. 2017;44(04):650-653+664.
23. 倪宁. 抗生素降阶梯治疗老年重症肺炎的临床疗效评价. *中国继续医学教育*. 2017;9(3)
24. 闫建华. 244 例老年肺炎的临床特点及治疗分析. *世界最新医学信息文摘（连续型电子期刊）*. 2017;17(A0):70-71. doi:10.19613/j.cnki.1671-3141.2017.100.046
25. 闫郑伟. 综合社区护理及家庭护理干预对老年吸入综合征的预防作用探讨. *系统医学*. 2017;2(24):137-139. doi:10.19368/j.cnki.2096-1782.2017.24.137
26. Li X, Blais JE, Wong ICK, et al. Population-based estimates of the burden of pneumonia hospitalizations in Hong Kong, 2011-2015. *European Journal of Clinical Microbiology and Infectious Diseases*. 04 Mar 2019;38(3):553-561.
27. 董莹, 许国章, 张良, 李宁, 林慧波. 基于区域卫生信息平台肺炎住院患者直接医疗费用分析. *社区医学杂志*. 2020;18(23):1621-1624. doi:10.19790/j.cnki.JCM.2020.23.14
28. 刘令初, 靳妍, 何寒青, et al. 2018－2019 年台州市老年人接种流感疫苗的成本效益. Benefit-cost ratio of influenza vaccination among elderly people of Taizhou city during the 2018－2019 season. *中国疫苗和免疫*. 2020;26(5)2021111182.
29. 王静, 王丽香, 魏丽娟. 应用临床路径精细化管理老年社区获得性肺炎住院患者抗菌药物合理应用的效果. *中华老年医学杂志*. 2020;39(6):622-626. doi:10.3760/cma.j.issn.0254-9026.2020.06.004
30. Li XZ, Jin F, Zhang JG, et al. Treatment of coronavirus disease 2019 in Shandong, China: a cost and affordability analysis. *Infectious Diseases of Poverty*. 2020;9(1):78.
31. 涂正波, 万刚凤, 肖红茂. 2017－2018 年南昌市城区流感病例经济负担和影响因素分析. Economic burden and influencing factors of influenza cases in urban area of Nanchang City,2017－2018. *现代预防医学*. 2021;48(1)2021177697.
32. 吴一峰, 李萍萍, 赵凤敏, 罗丽. 宁波市江北区≥60 岁老年人群接种流感疫苗后流感样疾病经济负担. Economic burden of influenza-like illness among≥60-year-old people after influenza vaccination in Jiangbei district of Ningbo city. *中国疫苗和免疫*. 2021;27(4)2021472678.
33. Lai X, Rong H, Ma X, et al. The Economic Burden of Influenza-Like Illness among Children, Chronic Disease Patients, and the Elderly in China: A National Cross-Sectional Survey. Research Support, Non-U.S. Gov't. *International Journal of Environmental Research & Public Health [Electronic Resource]*. 2021;18(12):10.
34. 徐英, 董碧蓉. 23 价肺炎球菌多糖疫苗预防老年人下呼吸道感染的效果考察. *中国计划免疫*. 2005;11(4):287-291. doi:10.3969/j.issn.1006-916X.2005.04.011
35. 麦华玉, 卢树标, 卢燕珊. 临床护理路径对老年人社区获得性肺炎的影响. *中国医药指南*. 2012;10(31):346-347. doi:10.15912/j.cnki.gocm.2012.31.084
36. 陈健. *上海地区流行性感冒流行规律与防控策略研究*. 博士. 复旦大学; 2013.
37. 程念, 付晓光, 汪早立. 新农合监测点参合老年人 7 个常见住院病种分析. *中国农村卫生事业管理*. 2013;33(03):241-244.

38. 张雅娟. 老年肺炎 122 例分析. *中国保健营养（中旬刊）*. 2013;(11):678-678.
39. 张晓羽. 老年风温肺热病中医临床路径的评价. *陕西中医*. 2015;(2):160-162. doi:10.3969/j.issn.1000-7369.2015.02.015
40. 甘语波. 老年性肺炎临床特点分析及临床诊治研究. *中国保健营养*. 2018;28(19):28-29. doi:10.3969/j.issn.1004-7484.2018.19.030
41. 孙聪. *青岛地区病毒性肺炎的流行病学研究及病原学分析*. 硕士. 青岛大学; 2019. https://d.wanfangdata.com.cn/thesis/ChJUaGVzaXNOZXdTMjAyMzAxMTISCUQwMTgwMjQ0NxoIM3Z6aHc3dHo%3D
42. 尹超男, 王海涛, 魏佳特, 赵琳, 曹务春. 河南省 1272 例新型冠状病毒肺炎疾病负担分析. Disease burden analysis of 1 272 COVID-19 cases in Henan province. *河南预防医学杂志*. 2021;32(5)2021448048.
43. 黄文姬. 综合社区护理及家庭护理干预对老年吸入综合征的预防作用研究. *齐齐哈尔医学院学报*. 2015;36(19):2960-2961.
44. 梁燕芳. 综合社区护理及家庭护理干预对老年吸入综合征的预防作用分析. *现代诊断与治疗*. 2015;26(04):948-949.
45. 李朝晖. 综合社区护理及家庭护理干预对老年吸入综合征的预防作用分析. *中国卫生产业*. 2016;13(17):196-198. doi:10.16659/j.cnki.1672-5654.2016.17.196
46. 那坤艳. 综合社区护理及家庭护理干预对老年吸入综合征的预防作用分析. *当代医学*. 2016;22(13):103-104.
47. 瞿玉仙. 综合社区护理及家庭护理干预对老年吸入综合征的预防作用研究. *中国保健营养*. 2016;26(11):400-400.
48. 谭星宇, 何权瀛, 王月珠, 赵学红. 362 例社区获得性肺炎患者住院费用调查. *中华医院管理杂志*. 2002;(07):32-35.
49. 董军, 曹秀堂, 亚春, 袁志军, 刘志敏, 许红民. SARS 住院病人医疗费用及影响因素分析. *中国卫生质量管理*. 2003;(05):48-50. doi:10.13912/j.cnki.chqm.2003.05.024
50. 肖峰, 陈博文, 武阳丰, 王月香, 韩德民, 北京市 SARS 医疗救治指挥中心科技攻关组. 北京市 SARS 临床诊断病例住院费用及影响因素分析. *中华流行病学杂志*. 2004;(04):43-47.
51. 王红军. 老年社区获得性肺炎住院患者 172 例临床分析. *重庆医学*. 2005;(09):1400-1401.
52. 王建华, 杜琳, 罗不凡, 刘伟佳, 潘冰莹. 广州市 SARS 病例经济负担研究. *华南预防医学*. 2006;(04):14-17.
53. 姚盛思. 老年肺炎患者家庭病床治疗 21 例结果分析. *基层医学论坛*. 2006;10(19):883-884. doi:10.3969/j.issn.1672-1721.2006.19.013
54. 关华, 黄君瑶, 杨哲, 李奕明. 广东省 1353 例 SARS 病人住院费用分析. *中国卫生统计*. 2007;(01):63+65.
55. 程真顺, 杨炯, 叶燕青. 老年社区获得性肺炎临床分析. *武汉大学学报(医学版)*. 2011;32(06):843-845+856. doi:10.14188/j.1671-8852.2011.06.011
56. 闫翔, 范慧民, 李钰, 沈桂林, 陈曦. 老年重症肺炎抗生素降阶梯治疗及比阿培南的应用. *中国医院用药评价与分析*. 2011;11(12):1126-1128.
57. 李志杰, 张伟华. 中西医结合治疗老年重症肺炎疗效观察. *中国社区医师（医学专业）*. 2012;14(36):140. doi:10.3969/j.issn.1007-614x.2012.36.131
58. 陈志燕, 金桂芳. 循证护理模式在老年社区获得性肺炎患者中的应用. *航空航天医学杂志*. 2013;24(12):1580-1582.
59. 顾建英, 蔡映云, 叶晓芬. 我院 2010-2012 年老年社区获得性肺炎患者抗菌药物应用分析. *中国药房*. 2013;24(22):2044-2046.

60. 李宏, 杨岚, 石志红. 肺部感染评分对老年重症肺炎患者抗菌药物选择的干预及对预后影响. *中华医院感染学杂志*. 2013;23(12):2814-2815+2825.
61. 李秀荣, 葛晓励, 王红阳, et al. 机械振动排痰在老年社区获得性肺炎中的临床应用. *河北联合大学学报（医学版）*. 2013;15(1):73-74. doi:10.3969/j.issn.1008-6633.2013.01.049
62. 张益辉, 牛忆军, 张欢. 降钙素原在社区获得性肺炎诊治中的价值. *临床肺科杂志*. 2013;18(7):1195-1196. doi:10.3969/j.issn.1009-6663.2013.07.016
63. 钱玉英, 冯明, 李耘. 老年非重症社区获得性肺炎临床路径实施的效果分析. *中国老年学杂志*. 2014;34(11):3152-3153.
64. 王玉梅, 刘博. 复方清金化痰汤联合阿奇霉素治疗老年社区获得性肺炎效果评价. *社区医学杂志*. 2014;12(05):1-4.
65. 张晓辉, 王张锋, 霍有娟. 血清降钙素原在指导老年社区获得性肺炎患者抗生素治疗中的应用. *中国综合临床*. 2014;(7):703-705. doi:10.3760/cma.j.issn.1008-6315.2014.07.011
66. 陈妍, 赵先彬. 麻杏石甘汤加味与抗生素治疗老年获得性肺炎疗效观察. *中国急救医学*. 2015;35(z2):380-381. doi:10.3969/j.issn.1002-1949.2015.z2.240
67. 纪颖, 安晓杰, 邓赶飞. 老年社区获得性肺炎临床特点分析. *现代医药卫生*. 2015;(12):1845-1846. doi:10.3969/j.issn.1009-5519.2015.12.035
68. 刘美蓉, 王娜娜, 苏留超, 谭效锋. 老年人社区获得性肺炎临床治疗与预后分析. *中华老年医学杂志*. 2015;34(9):981-983. doi:10.3760/cma.j.issn.0254-9026.2015.09.015
69. 万秀英, 黄楚真, 石永久. 中西医结合强化气道护理干预老年社区获得性肺炎临床观察. *新中医*. 2015;47(12):248-250. doi:10.13457/j.cnki.jncm.2015.12.111
70. 曾小云, 李慧, 胡春林, 李欣, 禹移, 荆小莉. 老年人反复发生社区获得性肺炎的临床特征和危险因素分析. *中华全科医师杂志*. 2016;15(6):434-438. doi:10.3760/cma.j.issn.1671-7368.2016.06.008
71. 陈建新, 汤文英. 老年吸入综合征的社区居家护理干预效果评价. *现代诊断与治疗*. 2016;27(12):2321-2323.
72. 陈丽莉, 崔俪馨. 老年社区获得性肺炎的护理对策探讨. *中国农村卫生*. 2016;(18):45-45.
73. 胡循贵, 周卫文. 肺部感染评分在重症肺炎患者诊断及治疗中的应用价值分析. *内科*. 2016;11(02):273-275. doi:10.16121/j.cnki.cn45-1347/r.2016.02.39
74. 贾晓利, 李会东. 老年 2 型糖尿病合并社区获得性肺炎的临床特征. *河北医药*. 2016;(5):743-744. doi:10.3969/j.issn.1002-7386.2016.05.036
75. 李音, 姜红妮, 瞿介明. 老年、高龄老年和超高龄老年社区获得性肺炎的临床特征及预后对照分析. *老年医学与保健*. 2016;22(1):19-23. doi:10.3969/j.issn.1008-8296.2016.01.006
76. 邱小松. 肺部感染评分在重症肺炎患者诊断及治疗中的应用价值分析. *中国继续医学教育*. 2016;8(31):92-94. doi:10.3969/j.issn.1674-9308.2016.31.055
77. 叶敏, 杨杰. 痰热清注射液治疗社区获得性肺炎的临床疗效研究. *医学综述*. 2016;22(12):2463-2466.
78. 刘蕊. 抗生素降阶梯方案与传统抗生素方案治疗重症肺炎患者的临床疗效及安全性. *中国药物经济学*. 2017;12(03):38-40.
79. 徐玲, 卢滨, 徐红冰. 长期使用苯二氮革类药物对老年人社区获得性肺炎发展和预后的影响. *医学研究杂志*. 2017;46(7):137-140. doi:10.11969/j.issn.1673-548X.2017.07.034
80. 李伟涛, 孟娟, 王亚, 王晓旭, 李平. 血必净对老年重症社区获得性肺炎临床疗效与机制分析. *健康必读*. 2018;(17):27.
81. 丘韶校, 李元广, 何臣, 徐效峰. 肺部感染评分在重症肺炎患者诊断及治疗中的应用价值. *中国现代药物应用*. 2018;12(09):117-118. doi:10.14164/j.cnki.cn11-5581/r.2018.09.066

82. 张玉明. 优质护理在老年社区获得性肺炎患者中的应用及临床效果. *心理医生*. 2018;24(11):216.
83. 朱爱娥. 健康教育应用于老年支原体肺炎患者护理中的效果. *临床医学研究与实践*. 2018;3(30):195-196. doi:10.19347/j.cnki.2096-1413.201830087
84. 陈英, 秦志强, 叶溪, 张玉军, 莫志江. 广西 9 家三甲医院头孢类抗菌药物对社区获得性肺炎患者住院费用和住院天数的影响. *临床医药文献电子杂志*. 2019;6(55):170-172. doi:10.16281/j.cnki.jocml.2019.55.142
85. 郭玉梅, 庚俐莉, 舒姣洁, et al. 痰热清注射液联合参附注射液治疗老年重症肺炎的临床效果. *中国当代医药*. 2019;26(08):151-154.
86. 邱红侠. 综合社区护理及家庭护理干预对老年吸入综合征的预防作用. *中外女性健康研究*. 2019;(22):54-55.
87. 吴双胜, 马春娜, 张莉, et al. 北京市成年流感样病例就诊行为及其经济负担研究. *国际病毒学杂志*. 2019;26(2):82-86. doi:10.3760/cma.j.issn.1673-4092.2019.02.003
88. 尹慧洁. 临床路径在社区获得性肺炎诊治及护理中的实施. *医学食疗与健康*. 2019;(1):116-117.
89. 李义平, 魏碧莹, 赖富明, 张雅萱. 深圳市 105 例新型冠状病毒肺炎患者住院费用及结构分析. *中国医院管理*. 2020;40(03):42-44.
90. 吴学智. 肺炎患者住院费用的 DRGs 病例组合研究. *中国卫生统计*. 2020;37(02):235-238.
91. 肖祖华, 文雨, 付万升, 黄子祺. 抗生素降阶梯治疗对老年重症肺炎的疗效评价. *中国实用医药*. 2020;15(28):7-9. doi:10.14163/j.cnki.11-5547/r.2020.28.003
92. 刘杰. *上海市某三级综合性医院五个单病种疾病住院费用及影响因素研究*. 硕士. 上海财经大学; 2021.
93. 涂正波. *南昌市红谷滩新区流感经济负担及暴发疫情处置成本效益研究*. 硕士. 南昌大学; 2021.
94. 陈艳艳. 肺部感染评分在老年重症肺炎患者抗菌药物选择中的应用效果. *中国民康医学*. 2022;34(5):147-149. doi:10.3969/j.issn.1672-0369.2022.05.048
95. 胡婷婷, 龚怀宇, 窦丰满, et al. 成都市某定点医院新型冠状病毒肺炎患者住院费用影响因素. *中国病案*. 2022;23(05):78-81.
96. 李燕菊, 冯英, 周晶晶, et al. 新疆某医院老年社区获得性肺炎住院患者抗菌药物使用情况调查. *河北医药*. 2022;44(22):3496-3499,3503. doi:10.3969/j.issn.1002-7386.2022.22.035
97. 李育梅, 吴俊霞. 某市三甲传染病医院新型冠状病毒肺炎患者流行病学特征和住院费用分析. *中国病案*. 2022;23(01):46-49.
98. 任秀君, 陈俊君. 肺部感染评分联合血清降钙素原在指导老年重症肺炎患者合理使用抗生素中的应用研究. *实用医院临床杂志*. 2022;19(03):181-184.
99. 徐江奇, 黄小红, 肖婷. 肌少症对老年社区获得性肺炎患者预后的影响. *江西医药*. 2022;57(09):1104-1106.
